# The Effects of Aroma Foot Massage on Blood Pressure and Anxiety in Japanese Community-Dwelling Men and Women: A Crossover Randomized Controlled Trial

**DOI:** 10.1371/journal.pone.0151712

**Published:** 2016-03-24

**Authors:** Eri Eguchi, Narumi Funakubo, Kiyohide Tomooka, Tetsuya Ohira, Keiki Ogino, Takeshi Tanigawa

**Affiliations:** 1 Department of Public Health, Okayama University Graduate School of Medicine, Dentistry and Pharmaceutical Sciences, Okayama, Japan; 2 Department of Public Health, Juntendo University Graduate School of Medicine, Tokyo, Japan; 3 Department of Epidemiology, Fukushima Medical University School of Medicine, Fukushima, Japan; Kurume University School of Medicine, JAPAN

## Abstract

**Objectives:**

The aim of this study was to investigate the effects of aroma foot massage on blood pressure, anxiety, and health-related quality of life (QOL) in Japanese community-dwelling men and women using a crossover randomized controlled trial.

**Methods:**

Fifty-seven eligible participants (5 men and 52 women) aged 27 to 72 were randomly divided into 2 intervention groups (group A: n = 29; group B: n = 28) to participate in aroma foot massages 12 times during the 4-week intervention period. Systolic and diastolic blood pressure (SBP and DBP, respectively), heart rate, state anxiety, and health-related QOL were measured at the baseline, 4-week follow-up, and 8-week follow-up. The effects of the aroma foot massage intervention on these factors and the proportion of participants with anxiety were analyzed using a linear mixed-effect model for a crossover design adjusted for participant and period effects. Furthermore, the relationship between the changes in SBP and state anxiety among participants with relieved anxiety was assessed using a linear regression model.

**Results:**

Aroma foot massage significantly decreased the mean SBP (*p* = 0.02), DBP (*p* = 0.006), and state anxiety (*p* = 0.003) as well as the proportion of participants with anxiety (*p* = 0.003). Although it was not statistically significant (*p* = 0.088), aroma foot massage also increased the score of mental health-related QOL. The change in SBP had a significant and positive correlation with the change in state anxiety (*p* = 0.01) among participants with relieved anxiety.

**Conclusion:**

The self-administered aroma foot massage intervention significantly decreased the mean SBP and DBP as well as the state anxiety score, and tended to increase the mental health-related QOL scores. The results suggest that aroma foot massage may be an easy and effective way to improve mental health and blood pressure.

**Trial Registration:**

University Hospital Medical Information Network 000014260

## Introduction

Mental stress and anxiety are major causes of hypertension and mortality from cardiovascular diseases. For example, the Whitehall II Study, a cohort study of 7268 British men and women, showed that perception of stress was associated with an increased risk of coronary heart disease and found that those with perceived stress had a double the risk of coronary heart disease than those without perceived stress [1]. Moreover, acute mental stress has been shown to trigger cardiac catastrophes such as acute myocardial infarction and sudden death [2]. According to Walker et al., each year 14% of deaths worldwide (approximately 8 million deaths) are ascribed to mental disorders such as anxiety and depression [3]. Furthermore, according to the Japanese Ministry of Health, Labour and Welfare, in 2014, more than 60% of workers reported feeling stress, anxiety, and worry. Therefore, it is important to reduce mental stress and anxiety to increase quality of life (QOL) and prevent cardiovascular diseases.

Although aromatherapy is often referred to as complementary and integrative medicine, it is one of the many ways to relieve mental stress. Aroma essential oils are generally inhaled or massaged into the skin, and the oil vaporizes and stimulates the olfactory system [4]. Consequently, according to a Cochrane review [5], essential oils have many effects including calming and de-stressing effects as well as promoting relaxation and sleep. For example, a previous study reported that inhalation of ylang-ylang, a frequently used aroma oil, decreased blood pressure (BP) in healthy men who participated in a randomized controlled trial (RCT) [6]. Similarly, an RCT crossover study conducted with 36 female high school students showed that, the stress levels in the intervention group decreased significantly compared to the placebo group after inhalation of aroma essential oils [7]. Furthermore, studies have shown that ambient odors of lavender and orange decreased anxiety and lightened mood in a dental office [8], and massages with aroma oils promoted skin absorption of the oils [9] stimulating blood and lymphatic circulation, improving the oxygen and nutrient supply, relaxing muscle tone, and relieving emotional stress [10]. One study found that receiving an aroma body massage once a week for 4 weeks and applying aroma cream on the arms, legs, and abdomen daily reduced BP among participants [10]. Similarly, an open, semi-comparative trial with 12 breast cancer patients showed that anxiety, as measured by the State-Trait Anxiety Inventory (STAI), was significantly reduced after a 30-minute aroma body massage by skilled therapists [11]. Moreover, aroma massages twice a week for 4 weeks improved prefrontal cortex dysfunction and mild depression in 5 patients with depression [12]. These findings indicated that aroma massage is associated with a more relaxed mental condition as well as decreased BP and anxiety.

In light of these findings, the number of studies that have assessed the effect of aromatherapy on BP and anxiety in healthy volunteers using RCT methods is limited although there are many studies concerning the efficacy of aromatherapy. Accordingly, the aim of this study was to evaluate the effects of self-administered aroma foot massages on BP and anxiety in Japanese community-dwelling men and women using a crossover RCT design.

## Materials and Methods

The protocol for this study and supporting CONSORT checklist are available as supporting information; see the S1 CONSORT Checklist, S1 Protocol, and S2 Protocol.

### Ethics Statement

Ethical approval was obtained from the Human Ethics Review Committee of the Ehime University Graduate School of Medicine (approval number 1401001). Written informed consent was obtained from all participants before the baseline examination, and this study conformed to the Declaration of Helsinki guidelines.

We confirmed that all ongoing and related trials for this intervention were registered. Because of inadequate information for registration, the date of registration was delayed until after the enrollment of participants.

### Inclusion and exclusion criteria

Men and women aged 20 to 70 who lived in or near Matsuyama, Ehime Prefecture, Japan were eligible for inclusion in this study. A 72-year-old woman who insisted on participating in this study was also included. Participants were excluded if they were pregnant, had tachycardia (heart rate [HR] ≥110 beats/min), bradycardia (HR ≤50 beats/min), severe hypertension (systolic BP [SBP] ≥180 mmHg, diastolic BP [DBP] ≥110 mmHg), severe arrhythmia, or cardiovascular disease. Those who had already participated in a similar study conducted in 2012 as well as those with a health condition prohibiting participation in this study were also excluded.

### Subjects

**Fig 1:**
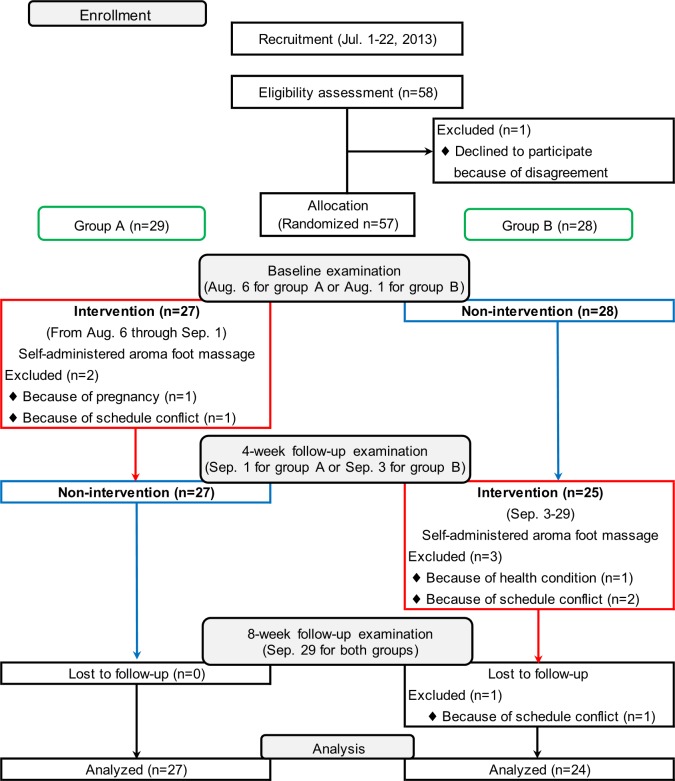
Flowchart and schedule of trial.

Fifty-eight participants (5 men and 53 women) aged 27 to 72 were eligible for inclusion. As the flowchart in Fig 1 shows, 1 woman was excluded because she declined to participate (n = 57), and the remaining 57 participants were randomized into 2 intervention groups (n = 29 and n = 28, respectively). One woman dropped out before the baseline examination because of pregnancy and 1 woman dropped out because of a scheduling conflict (n = 27 and n = 28, respectively). In addition, 3 women dropped out before the 4-week follow-up examination; 1 because of a health condition, and 2 because of a scheduling conflict (n = 27 and n = 25, respectively). One woman dropped out before the 8-week follow-up examination because of a scheduling conflict (n = 27 and n = 24, respectively). No harm or side effects occurred throughout the trial.

### Study design

This study was a crossover RCT. Participants were recruited between July 1 and July 22, 2013 through flyers and newspaper advertisements. Information regarding sex, age, and SBP level was obtained from the study application. Participants were randomly divided into 2 groups stratified by sex, age (<50 years old and ≥50 years old), and SBP (<130 mmHg and ≥130 mmHg). The allocation of the 2 groups was carried out randomly using random numbers ranging from 0 to 1 with a cutoff value of 0.5. An administrative staff member handled participant enrollment and automatically allocated participants into 1 of the 2 groups based on the random numbers generated by Excel. Participants were allocated into group A (n = 29) or group B (n = 28) using a 1:1 ratio. The baseline examination was held on August 1 for group B and August 6 for group A. The first follow-up examination (4-week follow-up) was held on September 1 for group A and on September 3 for group B. The second follow-up examination (8-week follow-up) was held on September 29 for both groups. All examinations and interventions were held at a fitness club in Matsuyama, Japan. The intervention sessions were conducted between August 6 and September 1, 2013 for group A, and between September 3 and September 29, 2013 for group B. The study schedule is shown in Fig 1. The sample size was determined by a power calculation based on previous results regarding hypertension in middle-aged women. These results indicated that the mean BP decreased by 15 mmHg after aroma massage [10]. Therefore, given that participants in our study were not patients, we presumed that the decrease in BP would be less than in the aforementioned study and, thus, assumed a decrease of 10 mmHg. With the standard deviation, significance level, and number of subjects in the study at 12, 0.05, and 51, respectively, the power calculation in this study using a crossover design was 82.3%.

### Interventions

After a 10-minute footbath, participants performed an aroma foot massage for 45 minutes on themselves under the supervision of a well-trained instructor. First, participants put the oil on their hands and inhaled the fragrance. Next, they applied the oil to their legs and massaged them with sweeping and gliding strokes from their thighs to their toes using their fingers and the palms of their hands. Participants also stimulated their acupuncture points at a moderate pressure and speed. After the massage, participants laid on their back and relaxed for 5 minutes. The room temperature and humidity were 38˚C and 65%, respectively, (standard temperature in many Japanese hot studios and considered the optimum temperature to promote perspiration during exercise). Participants were allowed to drink water and about 40 mL of hot ginger water during the process. All participants performed these procedures 3 times a week for 4 weeks (a total of 12 times) during the intervention period. These procedures were conducted on Tuesdays, Thursdays, and Sundays, and participant attendance was recorded. Participants who could not participate on the intervention days were allowed to participate on other days during the same week. The aroma oil used in this trial was blended for relaxation as well as mental and physical health by an aromatherapy specialist. Lavender, chamomile, sandalwood, ylang-ylang, and marjoram were blended with jojoba (a carrier oil) and preserved at room temperature for use.

### Measurements

#### Blood pressure and heart rate

After 5 deep breaths, SBP, DBP, and HR were measured on the right arm using an automatic sphygmomanometer (BP-103i II, Omron Colin, Kyoto, Japan) with participants in the seated position. After the first BP measurement, participants took 2 deep breaths, and a second measurement was taken. The mean value of the 2 measurements was used for the analyses.

#### State anxiety

The state anxiety and psychological stress scores were evaluated by the Japanese version of the STAI (STAI–JYZ) [13,14]. The STAI is one of the most popular measures of anxiety [15] and has been used worldwide because of its validity and reliability [13,16]. The anxiety reflects ephemeral feelings (such as nervousness, worry, and tension) associated with activation of the autonomic nervous system and indicates a participant’s recognition of stressors in the environment at a particular moment [15]. The STAI-JYZ includes 20 questions, and participants answered each question using a 4-point scale (not at all, somewhat, moderately so, or very much so) resulting in a score ranging from 20 points to 80 points. A higher score indicates greater anxiety. As described in the STAI-JYZ manual, which was referred to previous research papers [13], scores of state anxiety were divided into the following 5 groups: group 1 (<35 points), group 2 (35–45 points), group 3 (46–54 points), group 4 (55–64 points), and group 5 (≥65 points). These scores were categorized as low anxiety (groups 1 and 2) and high anxiety (groups 4 and 5). In previous research, low anxiety was defined as 45 points or below. Therefore, we classified participants with a state anxiety score of greater than 45 points as having anxiety. We also calculated the change in the state anxiety score (values after the intervention—values before the intervention). Finally, based on the change in the state anxiety score, we divided participants into the following 2 groups using the median in intervention periods: 1) people with relieved anxiety (i.e., a change of less than -3) and 2) people without relieved anxiety (i.e., a change of -3 or more).

#### Health-related QOL

Health-related QOL was measured by the 8-Item Short-Form Health Survey (SF-8) [17,18]. The SF-8 is the reduced version of the Medical Outcomes Study 36-Item Short-Form Health Survey, which has been used for evaluating health conditions worldwide. The Japanese version of the SF-8 was translated by Fukuhara et al. after a 2002 national survey (n = 1000) established the Japanese standards. The SF-8 has been used widely because of its reliability [17]. The instrument includes 8 questions assessing the following domains: physical functioning, role physical, bodily pain, general health perception, vitality, social functioning, role emotional, and mental health. Each response uses a 5- or 6-point scale and is normalized using the national standards. A score of 50 points represents the average Japanese standard, and higher scores indicate a better QOL. The physical component summary (PCS) encompasses the first 4 domains (physical functioning, role physical, bodily pain, and general health perception), while the mental component summary (MCS) encompasses the latter 4 domains (vitality, social functioning, role emotional, and mental health).

#### Lifestyle and physical examinations

Participants answered questions concerning lifestyle and behaviors such as habitual alcohol intake, smoking status, habitual exercise, optimism, subjective stress, stress relief methods, and satisfaction of sleep.

In the physical examination, we measured participants’ weight with bioelectric impedance analysis using an InBody 730 (InBody Japan Inc., Tokyo, Japan). The body mass index was calculated as weight/height (kg/m^2^).

### Statistical analysis

Mean values of the baseline characteristics for group A and B were calculated and compared using an unpaired two-tailed t-test or chi-square test. The intervention effects of aroma foot massage on SBP and DBP, state anxiety, health-related QOL, and the proportion of participants with anxiety were analyzed using a linear mixed-effect model for a crossover design adjusted for participant and period effects (combining group A and B). We also calculated the intervention effects stratified by age group (i.e., <50 years old and ≥50 years old) and changes in the SBP and state anxiety values (after intervention—before intervention). The relationship between the 2 values among participants with relieved anxiety was assessed and shown graphically using a linear regression model. SAS statistical software version 9.4 (SAS Institute Inc., Cary, NC, USA) was used for analyzing the data. *P*-values less than 0.05 were considered significant.

## Results

### Characteristics of participants at baseline

Participant characteristics at baseline are shown in Table 1. Participants were predominantly female (90.9%) with a mean age of 48.9 years, body mass index of 21.6 kg/m^2^, SBP of 107.6 mmHg, DBP of 68.5 mmHg, HR of 71.0 beats/min, state anxiety score of 40.9, SF-8 PCS score of 48.8, and MCS score of 46.0. There were no differences between group A and B at baseline.

**Table 1:** Baseline characteristics of participants.

Baseline variables		Group A (n = 27)	Group B (n = 28)	P value	Total (n = 55)
Women, %		88.9	92.9	0.61	90.9
Age, years		49.0±13.6	48.8±11.4	0.95	48.9±12.4
Body Mass Index, kg/m^2^		21.0±2.5	22.1±3.2	0.16	21.6±2.9
Systolic blood pressure, mmHg		108.1±13.3	107.1±14.6	0.79	107.6±13.8
Diastolic blood pressure, mmHg		69.2±10.9	67.9±10.2	0.65	68.5±10.5
Heart rate, beats/min		73.9±11.8	68.2±10.4	0.06	71.0±11.4
Habitual alcohol intake, %		40.7	57.1	0.22	49.1
Smoking status, %		3.7	0.0	0.30	1.8
Habitual exercise, %		25.9	21.4	0.69	23.6
Optimistic, %		63.0	53.6	0.48	58.2
Subjective stress, %		22.2	14.3	0.45	18.2
Employing stress relief method, %		66.7	60.7	0.65	63.6
Satisfaction of sleep, %		55.6	57.1	0.91	56.4
State Anxiety (STAI) score		41.1±11.2	40.6±10.0	0.88	40.9±10.5
SF-8 Health Survey Scoring					
	Physical component summary	48.4±7.1	49.3±7.6	0.66	48.8±7.3
	Mental component summary	44.7±8.3	47.2±7.1	0.24	46.0±7.7
	Physical functioning	49.1±5.5	49.8±4.6	0.64	49.5±5.0
	Role physical	47.6±6.1	49.2±5.3	0.31	48.4±5.7
	Bodily pain	48.7±8.6	51.7±9.5	0.23	50.2±9.1
	General health perception	48.4±6.7	48.2±7.7	0.92	48.3±7.1
	Vitality	48.0±7.3	49.1±6.8	0.55	48.5±7.0
	Social functioning	45.0±9.0	48.3±8.7	0.17	46.7±8.9
	Role emotional	47.2±6.4	49.9±4.9	0.09	48.5±5.8
	Mental health	45.7±6.9	47.6±6.5	0.29	46.6±6.7

Values are means ± standard deviation and ratios. *P* values indicate the significance of the differences between group A and B. STAI, State-Trait Anxiety Inventory; SF-8, the 8-Item Short Form Health Survey.

### Blood pressure

The mean SBP and DBP values at the baseline, 4-week follow-up, and 8-week follow-up examinations in both group A and B as well as the intervention effects of aroma foot massage on BP are shown in Table 2. The SBP and DBP values significantly decreased after the intervention. The SBP of group A was 108.1 mmHg before and 107.0 mmHg after the intervention, while for group B, it was 114.1 mmHg before and 110.0 mmHg after the intervention (*p* = 0.02). Similarly, the DBP for group A was 69.2 mmHg before and 67.3 mmHg after the intervention, while for group B, the DBP was 70.5 mmHg before and 68.8 mmHg after the intervention (*p* = 0.006). An intervention effect on DBP was found in the older age group (group A: before = 74.2 mmHg and after = 71.8 mmHg; group B: before = 76.3 mmHg and after = 73.7 mmHg; *p* = 0.01), but not in the younger age group. There were no significant changes in HR.

**Table 2:** Average blood pressure and mental health scores for the baseline, 4-week and 8-week follow-up examinations in group A and B.

			Examination			Changes		
Variables		Intervention group	Baseline	4-week follow-up	8-week follow-up	Intervention period	Non-intervention period	P value
Number		A	27	27	27	27	27	
		B	28	25	24	24	25	
Systolic blood pressure, mmHg		A	108.1±13.3	107.0±13.0	109.5±13.4	-1.07	2.43	0.02
		B	107.1±14.6	114.1±16.4	110.0±16.3	-3.52	6.20	
Diastolic blood pressure, mmHg		A	69.2±10.9	67.3±10.9	69.3±9.8	-1.87	2.00	0.006
		B	67.9±10.2	70.5±9.9	68.8±8.8	-1.38	2.18	
Heart rate, beats/min		A	73.9±11.8	72.7±9.6	72.9±10.7	-1.26	0.22	0.15
		B	68.2±10.4	72.8±10.2	76.1±10.5	3.40	4.40	
State Anxiety (STAI) score		A	41.1±11.2	38.0±9.4	40.4±10.0	-3.04	2.41	0.003
		B	40.6±10.0	40.0±9.2	35.0±9.5	-5.17	-0.16	
SF-8 Health Survey Scoring								
	Physical component summary	A	48.4±7.1	49.6±5.9	49.7±5.2	1.20	0.13	0.72
		B	49.3±7.6	50.7±4.8	51.4±5.2	0.53	1.67	
	Mental component summary	A	44.7±8.3	49.5±6.3	48.4±6.8	4.81	-1.15	0.088
		B	47.2±7.1	47.3±5.0	49.0±5.1	1.63	-1.09	
	Physical functioning	A	49.1±5.5	50.3±4.6	48.9±4.8	1.20	-1.44	0.11
		B	49.8±4.6	49.9±4.3	50.9±4.7	0.91	0.21	
	Role physical	A	47.6±6.1	49.8±4.6	49.8±4.0	2.19	0.03	0.98
		B	49.2±5.3	50.4±3.6	50.5±5.2	-0.22	0.88	
	Bodily pain	A	48.7±8.6	51.7±7.1	51.7±7.1	3.01	0.00	0.80
		B	51.7±9.5	53.4±6.9	53.0±8.2	-0.65	1.83	
	General health perception	A	48.4±6.7	50.9±8.1	51.4±7.3	2.53	0.45	0.15
		B	48.2±7.7	50.0±7.4	53.1±5.9	3.46	1.32	
	Vitality	A	48.0±7.3	51.3±5.9	51.1±6.6	3.33	-0.22	0.05
		B	49.1±6.8	50.3±5.9	53.1±4.6	2.98	0.77	
	Social functioning	A	45.0±9.0	49.9±7.1	49.2±6.3	4.93	-0.74	0.26
		B	48.3±8.7	48.7±7.2	50.6±6.7	1.11	-0.42	
	Role emotional	A	47.2±6.4	50.3±5.1	50.0±3.8	3.12	-0.30	0.94
		B	49.9±4.9	50.2±3.5	49.9±6.3	-0.56	-0.60	
	Mental health	A	45.7±6.9	50.4±5.8	48.5±8.3	4.70	-1.83	0.02
		B	47.6±6.5	47.8±5.3	49.8±5.7	2.05	-0.59	

Data are represented as means ± standard deviation and mean change. *P* values indicate the intervention effects of changes in the intervention and non-intervention periods (combining group A and B) analyzed using a linear mixed-effect model. Changes in the intervention period were based on the 4-week follow-up—baseline examinations of group A and the 8-week follow-up—4-week follow-up examinations of group B. Changes in the non-intervention period were based on the 8-week follow-up—4-week follow-up examinations of group A and the 4-week follow-up—baseline examinations of group B.

STAI, State-Trait Anxiety Inventory; SF-8, the 8-Item Short Form Health Survey.

### State anxiety and health-related QOL

The mean state anxiety and health-related QOL values at the baseline, 4-week follow-up, and 8-week follow-up examinations as well as the intervention effects of aroma foot massage on these factors are shown in Table 2. There were significant improvements in the state anxiety scores (group A: before = 41.1 and after = 38.0; group B: before = 40.0 and after = 35.0; *p* = 0.003). In addition, there was significant elevation in the score for mental health of health-related QOL (group A: before = 45.7 and after = 50.4; group B: before = 47.8 and after = 49.8; *p* = 0.02). In addition, the MCS and the score for vitality showed increases although they were not statistically significant. The MCS in group A was 44.7 before the intervention and 49.5 after, while the score in group B was 47.3 before and 49.0 after the intervention (*p* = 0.088). The score for vitality in group A was 48.0 before the intervention and 51.3 after, while the score in group B was 50.3 before and 53.1 after the intervention (*p* = 0.05).There were also no significant increases in the other domains of health-related QOL. After stratification by age group, the results indicated an intervention effect on state anxiety in the younger group (group A: before = 42.2 and after = 37.7; group B: before = 38.5 and after = 32.8; *p* = 0.004), but not the older group.

The proportion of participants with anxiety after the intervention as well as the intervention effect on state anxiety are shown in Table 3. There were significant decreases in the proportion of participants with anxiety (group A: before = 40.7% and after = 25.9%; group B: before = 40.0% and after = 16.7%; *p* = 0.003).

**Table 3:** Proportion of participants with anxiety at baseline, 4-week and 8-week follow-up examination in group A and B.

		Examination			
State anxiety score	Intervention group	Baseline	4-week follow-up	8-week follow-up	P value
≥ 45, %	A (n = 11)	40.7	25.9	40.7	0.003
	B (n = 11)	39.3	40.0	16.7	

The *p* value indicates the intervention effect between the intervention and non-intervention periods (combining group A and B) analyzed using a linear mixed-effect model.

### The relationship between changes in SBP and state anxiety

**Fig 2:**
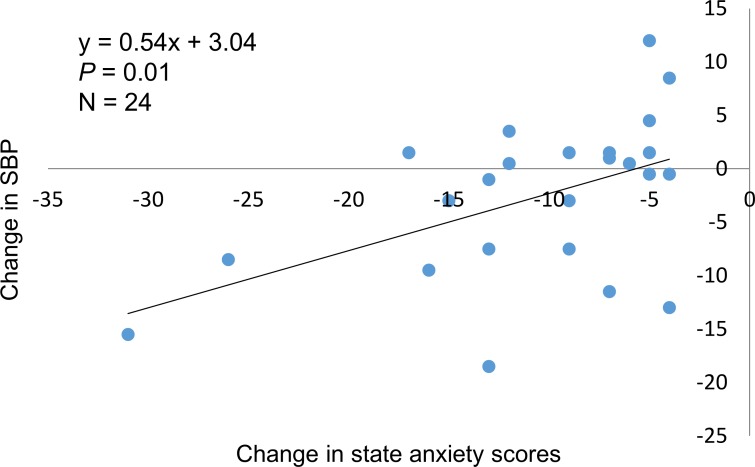
The relationship between changes in SBP and state anxiety in participants with relieved anxiety. The change in SBP had a significant and positive correlation with the change in state anxiety (*p* = 0.01) among participants with relieved anxiety (changes in state anxiety score <-3) after the intervention.

The relationship between changes in SBP and state anxiety in participants with relieved anxiety is shown in Fig 2. The change in SBP had a significant and positive correlation with the change in state anxiety (*p* = 0.01, n = 24).

## Discussion

The present study demonstrated that 12 self-administered aroma foot massage sessions over 4-week period significantly improved SBP, DBP, state anxiety, and mental health-related QOL among Japanese community-dwelling men and women. Self-administered aroma foot massage may be an effective way to improve mental health and BP.

A previous study has indicated that practitioner-conducted aroma body massages administered as a total of 5 sessions once a week for 4 weeks in addition to applying aroma cream on the arms, legs, and abdomen daily while also avoiding excessive exercise and dieting, decreased SBP by 15 mmHg in 28 middle-aged hypertensive women [10]. In our study, participants applied aroma oil on the legs 12 times over 4 weeks resulting in SBP and DBP reductions of 2 mmHg on average. Although the reductions of BP in our study were smaller than in other studies, this could be because most of the participants had a normal BP at baseline (the average SBP and DBP at baseline was 107.6 mmHg and 68.5 mmHg, respectively) and the intervention methods were different, which makes comparisons between the studies difficult. Our intervention may be easier and less expensive for the public. Other non-medical treatments that prevent hypertension, such as changes to an unhealthy diet, reducing excessive energy intake, increasing physical activity, and decreasing tobacco use [19], are not easy to maintain [20]. In contrast, our trial suggested that performing self-administered aroma foot massages is easy and may improve BP more than other interventions that require participants to make a lifestyle change.

Furthermore, to stimulate the olfactory system, we included lavender, ylang-ylang, marjoram, sandalwood, and chamomile in the aroma oil blend. Lavender relaxes the autonomic nervous system [21], calms and relaxes the level of emotion [22], and alleviates insomnia [9], while ylang-ylang decreases BP and HR [6], and reduces depression and stress [23]. Accordingly, reductions in BP and anxiety levels after our intervention might be because of lavender and ylang-ylang. In addition, the effects of linalyl acetate and linalool, which are major components of lavender and ylang-ylang essential oils [24,25], have been reported to affect the autonomic nervous system, especially the vagal tone [26,27], and decrease BP [27]. Therefore, the reductions in SBP and DBP in this study may be because of the activation of the vagal tone by linalyl acetate and linalool, thus making them beneficial depressors of BP and anxiety. Furthermore, some studies have shown that marjoram reduces BP in rats [28] and improves insomnia and sleeplessness through sedative effects [9]. In our study, improvements in SBP and DBP might also be associated with the effects of marjoram. In addition, the sedative effects of sandalwood extracts [29–31] and the use of chamomile, which has been shown to improve anxiety [32] and subjective sleep [33], and has antidepressant [34] and anxiolytic effects [35], may have improved anxiety scores among participants. Thus, the combination of various oils may have influenced relaxation, and improved BP and anxiety levels in this study. Furthermore, the combination of aromatherapy with massage therapy might improve BP and mental health through promoted absorption of the aroma oil by the skin and enhanced the calming and de-stressing effects through stimulation of the olfactory system [9].

We aimed to assess the effects of self-administered aroma foot massages on anxiety. Imanishi et al. showed that state anxiety was reduced after an aroma massage intervention among breast cancer patients [11] and Bagheri-Nesami et al. reported that anxiety was decreased after a foot reflexology massage intervention among 80 patients who underwent coronary artery bypass graft surgery [36]. Similarly, the average state anxiety scores and the proportion of participants who had state anxiety in this study decreased after the intervention, supporting results from previous studies. In addition, our study focused on community-dwelling participants, not hospitalized patients, and revealed the effectiveness of aroma massages in reducing anxiety among this population. Furthermore, Au et al. found that anxiety following surgery was relieved by acupressure in 5 RCT studies [37], and this may help explain the reduction of state anxiety scores after aroma foot massage in this study. In other words, the reduction found in this study might be partially contributable to stimulation of acupuncture points as well as aroma foot massage. Moreover, an RCT conducted by Varney and Buckle found that inhaling aromas 3 times a day for 3 weeks reduced mental exhaustion among 13 women and 1 man with mental exhaustion or moderate burnout [38]. Similarly, vitality increased after this intervention. In this study, the changes in SBP and state anxiety scores improved in participants whose anxiety was relieved after the intervention. As some other research has also shown that BP decreased on reducing anxiety [39,40], we presume that the primer change was reduction of anxiety followed by decrease of blood pressure. Thus, aroma foot massages may be efficacious in improving BP through reducing anxiety and improving the mental condition. Finally, an intervention effect was found on state anxiety in the younger group and on DBP in the older group reflecting effect differences according to age group.

This study has several strengths. First, to our knowledge, this was the first study to reveal positive effects of aroma massage on BP and anxiety in community-dwelling men and women (i.e., not ambulatory patients or inpatients) using a crossover RCT. Second, the aroma foot massage intervention could be easy to implement because it is easily conducted at home by the individual.

However, there are also some limitations to this study. First, because participants applied for the study themselves through flyers and newspaper advertisements and the trial schedule was during weekdays, they may have been more health-oriented than the general population and predominantly women who were interested in aroma foot massage, although the age and range were wide. Therefore, we may have underestimated the size of the effect and we need to consider the difference in the influence of specific age categories or sex when this intervention is implemented publicly. Second, it was difficult to differentiate the effects of the aromatherapy from the effects of the massage therapy; however, we hypothesized that a combination of aroma and massage therapy may have increased the effectiveness of the intervention. Finally, our study lacked a washout period before and after the 4-week follow-up. In group A, the average BP decreased after the intervention period but returned to baseline levels after the 8-week follow-up. However, the effect of the lack of a washout period was kept to a minimum because we adjusted for participant and period effects using the RCT study design.

In conclusion, our study showed that a self-administered aroma foot massage intervention conducted 3 times per week for 4 weeks improved BP, anxiety, and health-related QOL. Self-administered aroma foot massage may be easier to administer and may be an effective way to increase mental health and improve BP.

## Supporting Information

S1 CONSORT Checklist CONSORT checklist. (DOC)

S1 Protocol Trial protocol. Study protocol in English.
(DOCX)

S2 Protocol Trial protocol. Study protocol in Japanese.
(PDF)
