# Supplementary material for: The Effects of Aroma Foot Massage on Blood Pressure and Anxiety in Japanese Community-Dwelling Men and Women: A Crossover Randomized Controlled Trial
Source: PLoS One. 2016 Mar 24;11(3):e0151712. doi: 10.1371/journal.pone.0151712 (PMC4807074; doi:10.1371/journal.pone.0151712)
Supplement: S1 Protocol — Study protocol in English. (DOCX) [file pone.0151712.s002.docx]

**< TABLE OF CONTENTS >**

**Notice of Approval**

**Study application (summary)**

**Study protocol**

0. Title

1. Background and rationale

2. Purpose

3. Subjects

4. Study plan

5. Side effects of intervention

6. Assessments

7. Necessary measures to compensate the subjects for any injury occurring in the study

8. Ethics Statement

9. Cost allocation

**Study manual**

1. Study manual

2. Examination manual

**Additional Information**

1. Informed consent form

2. Flyer

**Study protocol**

0. Title

The mental and physical effects of aromatherapy massage on Japanese community-dwelling men and women: A crossover randomized controlled trial

1. Background and rationale

There are proven positive physiological and mental effects of aromatherapy. It has recently become popular and been used in many programs. However, there have been few studies about the effects of aromatherapy massage on blood pressure, anxiety, and mental health in healthy people over a long period of time.

2. Purpose

The aim of this study is to evaluate the mental and physical effects of aromatherapy massage on healthy Japanese people using a crossover randomized controlled trial.

3. Subjects

• Inclusion criteria

Men and women ranging in age from 20 to 70 years old who live in or near Matsuyama, Ehime Prefecture, Japan are eligible for inclusion in this study.

Participants are recruited through flyers and newspaper advertisements in Toon and Matsuyama, Ehime Prefecture.

• Exclusion criteria

Having severe illnesses.

- Severe arrhythmia or cardiovascular disease.
- Severe hypertension (systolic blood pressure (SBP) ≥180 mmHg, diastolic BP (DBP) ≥110 mmHg).
- Tachycardia (heart rate ≥110 beats/min) or bradycardia (heart rate ≤50 beats/min).
- Women who are pregnant.
- Those who participated in a similar study conducted in 2012.
- Those with health conditions are prohibited to participate in this study.

4. Study plan

• Randomization and allocation

Participants are randomly divided into two groups stratified by sex, age (<50 years old and ≥50 years old), and SBP (<130 mmHg and ≥130 mmHg). The allocation of the two groups is carried out using the method of minimization, and participants are allocated into the two groups using a 1:1 ratio. During the intervention period, participants participate in aromatherapy foot massage. The study is a non-blind trial.

• Sample size

The number of participants is 60 in 2013 and 80 in 2014, for a total of 140.

(With the standard deviation, significance level, and number of subjects in the study at 12, 0.05, and 51, respectively, the statistical power in this study was more than 80%).

• Intervention

After a footbath, participants perform an aromatherapy foot massage on themselves for 45 minutes under the supervision of a well-trained instructor at a room set to 38˚C and 65% humidity.

• Schedule

Participants perform an aromatherapy foot massage three times per week for four weeks (a total of 12 times) during the intervention period.

The baseline examination, first follow-up examination (four-week follow-up), and second follow-up examination (eight-week follow-up) are held, through which participants are evaluated physically and psychologically. After intervention in group A, the same intervention is done in group B. The same intervention study is held the next year.

| Group | Baseline examination | Intervention | First follow-up examination | Intervention | Second follow-up examination | After intervention |
| --- | --- | --- | --- | --- | --- | --- |
| A | Questionnaires  Examination | 3 times per week for a month (a total of 12 times) | Questionnaires  Examination | Non-intervention | Questionnaires  Examination | Presentation of results |
| B |  | Non-intervention |  | 3 times per week for a month (a total of 12 times) |  |  |
| Intervention: footbath and aromatherapy foot massage | | | | | | |

• Measurements

**Primary outcome measures**

1. Blood pressure (BP-103i II, Omron Colin)

Systolic and diastolic blood pressure, heart rate.

1. Function of autonomic nervous system (TAS9)

Heart rate Variability using an analyzer of the second derivative of photoplethysmogram (HRV: the clinical consequence of various influences of the autonomic nervous system on the heart beat).

1. Body composition analysis (InBody 730, InBody Japan Inc.)

Intracellular water, extracellular water (ECW), protein, minerals, body fat mass, skeletal muscle mass, muscle mass, lean body mass, weight, body mass index, percentage of body fat, Waist to Hip Ratio, segmental lean analysis, segmental lean, visceral fat level, waist circumference, ECW/total body water (TBW), nutrition evaluation, body balance, body strength, ideal weight, other parameters (obesity degree, body cell mass (BCM), bone mineral content (BMC), Basal Metabolic Rate (BMR), arm circumference (AC), Arm Muscle Circumference (AMC), waist, body composition history (10 times the accumulated results), and impedance of each segment/frequency.

1. Oxidative stress (oxidation-reduction measuring device, ARA! GENKI LL-001, Live & Love)

Measurement of the salivary oxidation-reduction potential values.

1. Ryodoraku acupuncture measurement (Neurometer MD-21, RYODORAKU RESEARCH INSTITUTE)

Measurement of electronic evaluation of the meridian system by measuring skin conductance at acupunctural points. The values of Ryodoraku reflect the conditions of the relative meridians and organs by analyzing and comparing their changes with a microelectrical current.

Secondary outcome measures

1. Lifestyles

Habitual exercise, food intake, habitual alcohol intake, smoking status, and sleep (questionnaires)

1. Subjective stress (questionnaires)
2. Health-related quality of life (8-Item Short-Form Health Survey [Sf-8] questionnaires)
3. Anxiety (State-Trait Anxiety Inventory [STAI] questionnaires)
4. Profile of Mood States ([POMS] questionnaires)

Six scale scores: Anger-Hostility, Confusion-Bewilderment, Depression-Dejection, Fatigue-Inertia, Tension-Anxiety, and Vigor-Activity.

• Discontinuous criteria

In any case where we encounter harmful information or comments about the study, there is a need to inform participants that the study will be stopped immediately.

5. Side effects of intervention

Adverse effects of the aroma that we use in aromatherapy foot massage have not before been reported. The examinations are minimally invasive, and complications have not before been reported.

6. Assessments

• Primary outcomes

①Blood pressure, ②function of the autonomic nervous system, ③body composition analysis, ④stress, ⑤Ryodoraku acupuncture measurement

• Secondary outcomes

①Lifestyles, ②stress, ③health-related quality of life, ④anxiety, ⑤profile of mood states

7. Necessary measures to compensate the subjects for any injury occurring in the study

• Compensation

Any injury caused by participating in the study is covered by the Nisshin Fire & Marine Insurance Co., Ltd. for a member of the fitness club. Participants who are not a member of the fitness club automatically are insured by the proponent of the study before participating in the study.

8. Ethics Statement

• Potential benefits and harm

The potential benefits are participation in the study and receiving the results at no cost. No harm or side effects should occur throughout the trial. Participants would be seen doctors immediately in case of any unexpected side effects during the trial.

• Selection of other treatments

Nothing in particular.

• Informed consent procedure

After an explanation of the study to subjects, their written informed consent is obtained in order to conduct the study.

• Publication of study outcomes

- We present the results to and inform participants individually.
- Only anonymous results are published, without mention of the participant's identity.

• Protection of privacy

The “linkable anonymizing method” is used to protect personal information.

(Personal information, such as names, is removed from the data and samples of subjects, and the data and samples are anonymized by assigning new codes or numbers instead. The correspondence table matching the subjects and these codes [numbers] is strictly protected to prevent it from being leaked). The correspondence table is recorded to an external memory medium (e.g., universal serial bus (USB) memory) by using a computer not connected to the network. The medium is strictly stored in a locked place.

9. Cost allocation

• Health insurance, copayment, exception treatment

There is no copayment required for participating in the study.

• Appearance fee

None.
