# Supplementary material for: The Effects of Aroma Foot Massage on Blood Pressure and Anxiety in Japanese Community-Dwelling Men and Women: A Crossover Randomized Controlled Trial
Source: PLoS One. 2016 Mar 24;11(3):e0151712. doi: 10.1371/journal.pone.0151712 (PMC4807074; doi:10.1371/journal.pone.0151712)
Supplement: S2 Protocol — Study protocol in Japanese. (PDF) [file pone.0151712.s003.pdf]

＜目次＞

臨床研究に関する決定通知書・・・・・・・・・・・・・・・・・・・・・・ 1

臨床研究申請書（概要）・・・・・・・・・・・・・・・・・・・・・・・ 2-3

臨床研究計画書・・・・・・・・・・・・・・・・・・・・・・・・・・・ 4-7

0. タイトル
1. 研究の背景、着想に至った根拠
2. 研究の目的
3. 対象
4. 研究計画
5. 薬物情報（副作用等）
6. 評価項目
7. 有害事象発生時の対応
8. 倫理的事項
9. 費用負担

研究マニュアル

1. マニュアル・・・・・・・・・・・・・・・・・・・・・・・・・・ 8-15
2. 検査概要・・・・・・・・・・・・・・・・・・・・・・・・・・・16-18

資料

1. 参加者への説明文書・・・・・・・・・・・・・・・・・・・・・・・19-25
2. ちらし・・・・・・・・・・・・・・・・・・・・・・・・・・・・・26-27

自主臨床研究様式08号

愛大医病倫1401001号
西暦2014年1月27日

臨床研究に関する指示・決定通知書

臨床研究責任者
公衆衛生・健康医学分野　教授
谷川武　　　　　　殿

愛媛大学医学部附属病院長
檜垣　實男　　印

2013年12月12日 に申請のあった本臨床研究について，臨床研究倫理審査委員会にて本院の基準に従い協議され，下記のとおり決定したので通知します．

記

臨床研究課題名	足浴と温熱環境におけるアロママッサージによるリラクセーション効果に関する心理的及び身体的影響についての比較研究
臨床研究代表者（研究組織名）	谷川武
臨床研究責任者	谷川武
臨床研究分担者	江口依里
臨床研究協力者	友岡　清秀
臨床研究期間	2014年2月1日　より　2015年3月31日　まで
予定症例数	140　症例
審査事項	✓ 臨床研究実施の可否<br>□ 臨床研究継続の可否<br>□ 臨床研究実施計画書の可否<br>□ 重篤な有害事象の発生<br>□ 新たな安全性に関する情報の入手<br>□ 逸脱に対する承認<br>□ その他（　　　　）
審査結果	承認する
承認番号	愛大医病倫　1401001　号
指示事項	特段なし
備考<br>右欄には直接入力してもよい．あるいは，印刷後に手書きで記入してもよい．	

本決定通知書に対して異議がある場合は，7日以内に研究協力課に文書で連絡してください．

6.00

受付番号

2013年12月12日

臨床研究申請書

愛媛大学医学部附属病院長　殿

診療科(部)名　公衆衛生・健康医学分野
診療科(部)長　印
臨床研究責任者　谷川武　印

下記のとおり臨床研究を申請します.

記

1. 臨床研究課題名<br>温熱環境におけるアロマママッサージによる心理的及び身体的影響についての無作為化比較試験	
2.研究区分＊	■介入研究　□介入研究以外の臨床研究　□適応外使用<br>□その他:
3.U-MINの登録	■登録予定　□登録不要
4.研究のデザイン	A.　■比較試験　□観察研究　□その他<br>1)　□blind trial　■open trial<br>2)　□プラセボあり　■プラセボなし
5. 臨床研究代表者名(研究組織として行う場合は組織名も記入)<br>谷川武	
6. 臨床研究責任者<br>谷川武	
7. 臨床研究の背景および目的/必要性<br>足浴やアロマテラピーに関する様々な研究が行われ、生理的・心理的な効果が明らかになってきている。そして、近年ではこれらの持つリラクセーション効果が注目され、民間のフィットネスクラブなどでも、足浴などの温熱療法やアロマテラピーが取り入れられている。そして、しばしばこれらの施設では、足浴やアロマテラピーは単独で利用者に提供されるものではなく、様々なプログラムの中で複合的に取り入れられている。しかしながら、これらの複合的な効果に関する研究は少ない。そこで、本研究では足浴と温熱環境におけるアロマママッサージが心身に及ぼす影響について比較研究を行い検討する。	
8. 研究方法<br>本研究の対象は、松山市の民間フィットネスクラブ「そらともりREN」の利用者で、同意の得られた20～70歳の男女とする。まず、参加同意が得られたものを、無作為に介入群と対照群の2群に分ける。介入の方法は、まず足浴で足を温めた後に、室温38度　湿度68%のホットスタジオでアロマを使ったフットマッサージを行うというものである。また、ホットスタジオでは、生姜湯を服用する。足浴からフットマッサージまでの所要時間は、約45分間である。。対象群は検査のみを受け、プログラムには参加しない。介入は、週1回を2か月、計8回行う。介入の第1回の実施前、および第8回の実施後に検査を行い、身体的、心理的評価を行う。評価方法には、①Inbody測定②血圧測定③心電図測定④ストレス検査(唾液検査によるコルチゾール測定)⑤生活習慣問診票⑥健康観連QOL(SF-8)質問紙⑦STAI質問紙⑧WHO SUBI質問紙を行う。	
9. 臨床研究分担者<br>江口依里	
10. 臨床研究協力者<br>友岡　清秀	
11A. 研究に使用する医薬品,医療機器等<br>Inbody730 (Biospace製)<br>血圧計(BP-103iII、UA-767PC)<br>酸化還元確認計アラ！元気　LL-001(ストレス検査)<br>TAS9　自律神経(ストレス)分析　加速度脈波測定器<br>ノイロメーターMD-21(良導絡測定)	

6.00　＊適応外使用の場合、すべての様式の「臨床研究」を「臨床使用」と読み替える。

臨床研究申請書

受付番号

11B *医薬品及び医療機器の場合に記入

a.研究に使用する医薬品及び医療機器の区分(複数選択可)

□国内未承認薬　□院内製剤　□既発売医薬品(適応外使用)
□既発売医薬品　□国内未承認医療機器　■医療機器
■その他()

b.医薬品及び医療機器の管理場所・管理者

□通常管理　□責任医師管理
□薬剤部試験薬管理　□ME機器センター管理
■その他()

12. 臨床研究期間　2014年2月1日　～　2015年3月31日

13. 予定症例数　140 症例

14. 費用区分　■研究費　□校費　□保険診療
□基準外医療費(特別医療費・研究医療費)　□該当せず
□その他:

15. 研究実施場所:　そらともりREN

16. 臨床研究責任者の経験
有

17. 医学倫理的配慮について

1) 安全性に対する配慮

本研究で実施するプログラムについて、こまでに副作用などの不利益は報告されていない。本研究において障害が発生した場合には速やかに対応し、必要な場合には治療を行う。

2) 説明と同意に関する配慮

本研究への参加は、本人の自由意思であり十分な説明の後、本人の同意の下に行われる。また、同意をしなくてもそれによる不利益は何ら被ることはなく、同意は本人の意思でいつでも撤回することができる。

3) その他

本研究により収集されたデータは、全て連結不可能匿名化により解析および保存される。本研究の結果が学会や論文などで発表される際には、個人が特定できない形にする。また、参加者が本研究の結果を知りたい場合、他の参加者の個人情報や本研究の独創性に支障がない範囲内で、方法や結果について資料を入手または閲覧することができる。

18. 備考

6.00

臨床研究計画書

0. タイトル

温熱環境におけるアロママッサージによる心理的及び身体的影響についての無作為化比較試験

1. 研究の背景、着想に至った根拠

アロマテラピーに関する生理的・心理的な効果が明らかになってきている。近年ではこれらのリラクセーション効果が注目され、様々なプログラムの中で利用者に提供されている。しかしながら、これらの長期的な効果、あるいは健常者を対象とした効果についての検討はほとんど行われていない。

2. 研究の目的

本研究では健常者を対象とし、温熱環境におけるアロママッサージが心身に及ぼす影響について比較研究を行い検討する。また、既存データについて、フィットネスクラブでの運動及びリラクセーション効果を検討することを目的とする。

3. 対象

・選択基準（年齢等）

松山市周辺の住民で、本研究の説明を受け、同意を得られた20～70歳男女。

民間のフィットネスクラブ「そらともり REN」ホームページ、または配布チラシに掲載する他、東温市、松山市にてもポスターを配布、掲示し広く募集する。

・除外基準

重篤な疾患を持つもの。

・心症などの循環器疾患を持つ者

・重度高血圧（収縮期血圧 180mmHg 以上、拡張期血圧 110mmHg 以上）を持つ者

・安静時の脈拍が、110 拍/分以上、または 50 拍/分以下の者

・重症不整脈をもつ者

・妊娠中の者

・その他運動を中止するべきと判断された者

4. 研究計画

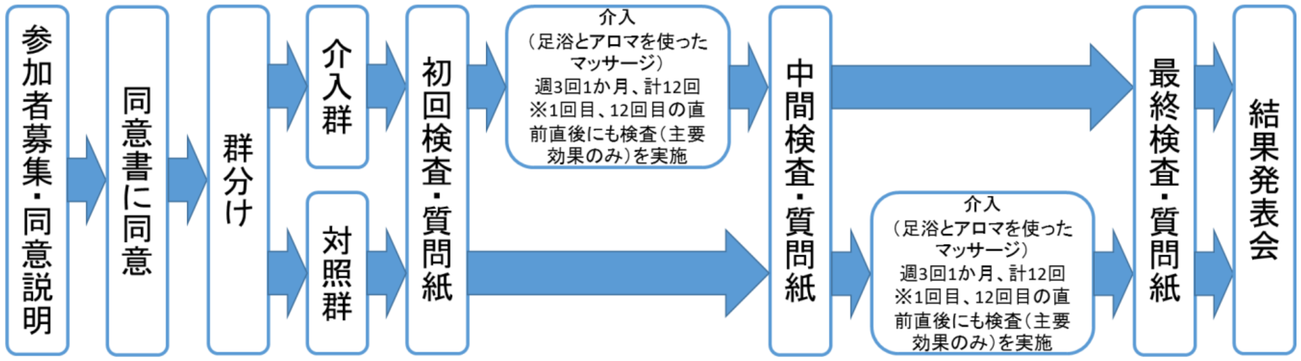

・群設定、盲検、非盲検、割り付け法

本研究に応募した者のうち、参加に同意した者について、性別、年齢、血圧値にて層別し、最小化法を用いて無作為に介入群と対照群の 2 群に１ ： １にて割り付ける。介入群はアロマを使ったフットマッサージのプログラムに参加し、対照群は、検査のみに参加する。本研究は盲検で実施することは不可能であるため、非盲検で実施する。

・予定参加人数

１年目 60 名、2 年目約 80 名、計 140 名　（A群、B群のそれぞれとコントロールの血圧値の差の平均を 10mmHg、標準偏差を 12 と設定し、検出基準を α=0.05 としたところ、1 グループあたり 30 人、計 80 人の参加にてパワーを 80%以上に維持することができる。自律神経機能についても）

・介入の方法

足浴で足を温めた後に、室温 38 度　湿度 68%のホットスタジオでアロマを使ったフットマッサージをインストラクターの指導により、自分で行う。所要時間は、約 45 分間である。

・スケジュール表

介入は、週３回を１か月、計１２回行う。

介入の第 1 回の実施前、および第 12 回の実施後に検査を行い、身体的、心理的評価を行う。また、介入群では第 1 回の介入直後および第 12 回の介入直前にも検査を行う。さらに、介入群終了後、クロスオーバーをさせ対照群にも同様の介入を行い、初回及び最終の介入で同様の検査を実施する。2 年目では、介入は週 3 回を 1 か月、計 12 回行う。介入の第 1 回の実施前、および第 12 回の実施後に検査を行い、身体的、心理的評価を行う。また、介入群では第 1 回の介入直後および第 12 回の介入直前にも検査を行う。さらに、介入群終了後、クロスオーバーをさせ対照群にも同様の介入を行い、初回及び最終の介入で同様の検査を実施する。

<table>
<tr><th></th><th>初回検査</th><th>介入期間</th><th>中間検査</th><th>介入検査</th><th>最終検査</th><th>実施後</th></tr>
<tr><td>介入群</td><td rowspan="2">質問紙
検査</td><td>週３回１か月
計 12 回※</td><td rowspan="2">質問紙
検査</td><td>介入なし</td><td rowspan="2">質問紙
検査</td><td rowspan="2">結果説明会</td></tr>
<tr><td>対照群</td><td>介入なし</td><td>週３回１か月
計 12 回※</td></tr>
<tr><td colspan="7">介入：足浴とアロマを使ったフットマッサージ
※1 回目実施後、12 回目実施前に検査（主要効果のみ）を行う</td></tr>
</table>

・検査項目等

主要効果の測定項目

生理指標：

①血圧測定（BP-103iII、UA-767PC）

最大／最小血圧、脈拍

②自律神経機能測定（TAS9）

加速度脈波測定器による HRV（Heart Rate Variability ： 心拍間変異分析）

③体成分分析（Inbody730 Biospace 製）

細胞内水分、細胞外水分、タンパク質、ミネラル、体脂肪量、骨格筋量、筋肉量、除脂肪量、体重、BMI、体脂肪率、ウエストヒップ比（WHR）、部位別筋肉量、部位別筋肉率、内臓脂肪レベル、腹囲、ECW/TBW(細胞外水分比)、栄養評価、身体バランス、身体強度、適正体重、体重調節量、脂肪調節量、筋肉調節量、フィットネススコア、研究項目（Obesity Degree、BCM、BMC、BMR、AC、AMC、Waist）、体成分累積結果（10 回測定結果）、部位別・周波数別インピーダンス提供

④ストレス検査（酸化還元確認計アラ！元気 LL-001）

口腔内に入れた綿棒に唾液をしみこませることにより、唾液の酸化還元度を測定する。

⑤東洋医学検査（MD-21）

ノイロメーターによる良導絡測定。

副次効果の測定項目

①生活習慣

運動習慣、食生活、飲酒、喫煙、睡眠（質問紙による）

②自覚的ストレス（質問紙）

③健康観連 QOL（SF-8 質問紙にて 8 つの下位尺度から測定）

④不安状態（STAI 質問紙にて状況不安、特性不安を測定）

⑤気分や感情の状態(POMS 質問紙にて「緊張」「抑うつ」「怒り」「活気」「疲労」「混乱」の 6 つの尺度から気分や感情の状態を測定)

・中止基準

本研究を実施する上で有害な情報が得られた場合、研究を中止し、速やかに対象者に連絡をする。

5. 薬物情報（副作用等）

本研究にて使用するアロマによるフットマッサージにて、これまでに副作用などの不利益は報告されていない。検査も侵襲性は低く、これまでに合併症などは報告されていない。

6. 評価項目

・主要エンドポイント、副次エンドポイント

本研究での主要エンドポイントは、①血圧測定 ②自律神経機能測定 ③体成分分析 ④ストレス検査 ⑤東洋医学検査とする。副次エンドポイントは、①生活習慣 ②自覚的ストレス ③健康観連 QOL ④不安状態 ⑤気分や感情の状態とする。

7. 有害事象発生時の対応

・補償等

研究参加時の事故にて障害が発生した場合、フィットネス会員であれば既に加入している日新火災海上保険の賠償責任保険に基づき、必要な場合には保険の範囲内にて治療する。また、フィットネス会員でない場合は、本研究参加前に同様の保険に加入する。

8. 倫理的事項

・期待される利益、不利益

本研究に参加する利益として、各種検査への参加、及びその結果の通知を無料で受けることがあげられる。本研究における副作用等は特にないと考えられ、不利益は特にはない。

・他の治療法の選択肢

特になし。

・同意取得方法

同意説明文および口頭により説明を行い、十分なインフォームドコンセントのもと同意書に自筆にて署名をする。

・研究結果の公表について

・個人の検査結果については結果説明会を行い個別に通知する。

・全体の結果については、集団として匿名化された解析結果のみを公表することとし、研究対象者個人が特定できないように配慮する。

・個人情報の保護

本研究で実施される検査のデータに関しては、連結可能匿名化を行い、個人情報を保護する。尚、連結可能匿名化の際には対応表を作成する。対応表はネットワークから切り離されたコンピューターを使用して、外部記憶媒体（USB など）に記録され、鍵をかけて厳重に保管する。

9. 費用負担

・保険診療、自己負担、基準外医療

本研究の検査などにおける対象者の個人費用負担は発生しない。但し、フィットネスクラブの会員費用については各自負担する。

・謝金の有無

特になし。

愛媛大学×そらともり REN

「身美温癒の身体的・心理的効果に関する研究」受付マニュアル

問い合わせ

・受付のためにRENのカウンターまできてもらう必要があると伝える
・7/22締切であることを伝える

来店・受付

・参加者の方がきたらまずチラシを見せてスケジュールの確認をする
•8/1の10時に来ることができるかを確認する
•OKであればファイルを見せながら研究の説明にうつる

概要説明

・説明ファイルを見せながら説明する
・参加希望となれば申込にうつる

申込

・申込書記入例はマニュアルp.2を参照
・受け付け順に受付番号を左上に記入する
・その後、太枠内を参加者に記入してもらう
・人数が多い場合は抽選になるということを説明する

測定

・３つの検査（in body、血圧、自律神経）を行う（詳細は【検査について】を参照）
・データの入力（入力方法は【参加者情報の入力】を参照）

抽選・発送

・抽選結果は発送をもってかえることを確認する
・遅くても7/29までに届く

【参加申込書例】

「身美温癒」の身体的・心理的効果に関する研究

参加申込書

受付番号	

受付順に番号を記入

申し込み日	2013年	月	日

太枠内をご記入ください

ふりがな		性別	男　女	年齢	歳
氏名		生年月日	西暦 年 月 日		
住所	〒　－				
連絡先	（　　）				

この３つの※を必ず確認する

※対象は抽選にて決定します。ご希望に添えないことがありますことをご了承下さい。
※コースのご希望には添えませんがご了承ください。
※抽選結果は発送をもってかえさせていただきます。

測定後に記入

検査項目	チェック	備考・結果			
Inbody	□	※結果用紙には必ず受付番号と氏名を記入			
自律神経機能測定	□	※TAS9の入力時には必ず受付番号と氏名を記入			
血圧測定	□	1回目	最高血圧 mmHg	最低血圧 mmHg	脈拍 回/分
		2回目	最高血圧 mmHg	最低血圧 mmHg	脈拍 回/分

※血圧測定は安静状態で5回深呼吸ののちに1回目を計測、さらに2回深呼吸ののちに2回目を計測します。

【検査について】

〇Inbody 検査

・結果用紙の左上には必ず**受付番号と氏名を必ず記入**する。

・測定後、結果用紙の**表面のみコピーをとり原本は参加希望者に渡す。**コピーは保存する。

〇血圧測定（血圧は２回測定する。）

※血圧測定前に次のことに注意する。

・背筋を伸ばして姿勢よく座る。

・カフの中心が心臓の高さになるようにする。

・リラックスして安静にする。

・身体を動かしたり、おしゃべりしたりしない。

〈測定方法〉※血圧計は REN のものを使用

①カフに**右腕**を通す。この時、できるだけ素肌に直接まくようにする。ただし、T シャツなどの薄い素材であればそのまま測定可能。右腕が難しい場合、左腕でも測定可能。

②**５回深呼吸**してもらい、スイッチを押す。（１回目）

③１回目の計測後、**２回深呼吸**してもらい、スイッチを押す。（２回目）

〈記録と保存〉

・結果が出たら申込用紙に１回目、２回目の**最高血圧、最低血圧、脈拍**を記録する。

・申し込み用紙はコピーをとり原本は参加希望者に渡す。コピーは保存する。

〇TAS9（自律神経検査）※詳細は P.4～8 を参照

用意するもの

１．TAS９（YKC　Corporation）①

２．パソコン（PC1）

３．プリンター（PC1）

４．延長コード

TAS9 の結果用紙についてはコピー不要。PC にデータが残ります。

【参加者情報の入力】

３つの測定がすべて終わったら参加者の情報を受付番号順にエクセルシートに入力する。その際、エクセルシートの見本を参照に、数字・記号は半角、その他は全角で入力する。**友人や家族での参加の場合は、必ず備考欄に記載する。**

〈TAS9 測定方法〉

準備

①大学から TAS9①とプリンタ―を REN に持っていく。

②TAS9、PC,プリンターをセットする。PC には無線受信用の USB をさす。

測定

①PC を起動させ、TAS9 が正しく接続されていることを確認する。

②TAS9 の電源を入れる。電源は少し長押し。

③PC のスタート画面（Windows マーク）から TAS9 を立ち上げる。

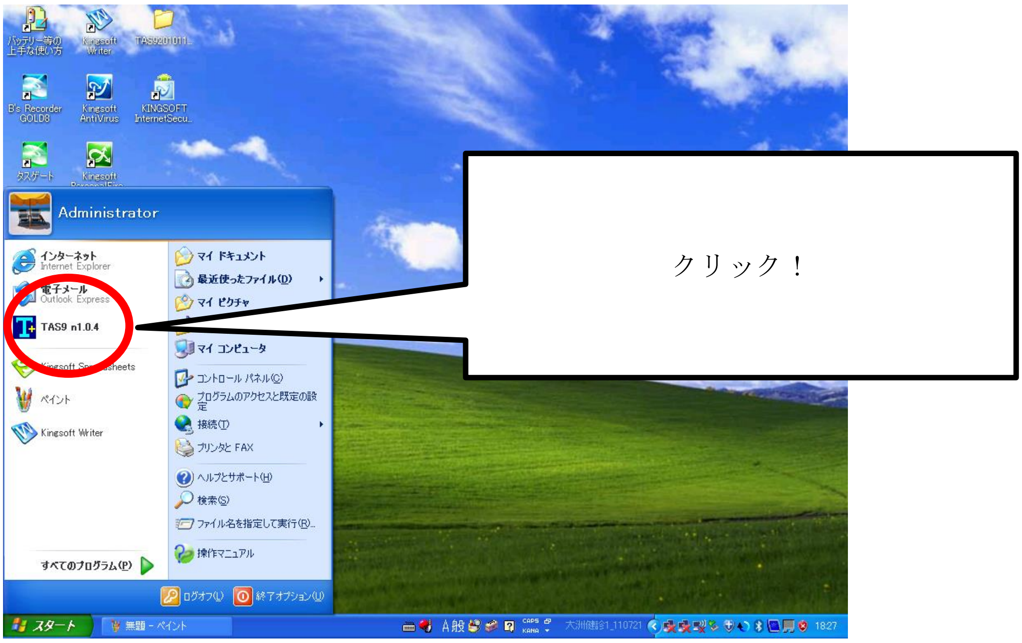

⑤TAS9 が起動したら、PC 画面の「HRV」を選択。さらに「F1」を選択して登録画面を出す。

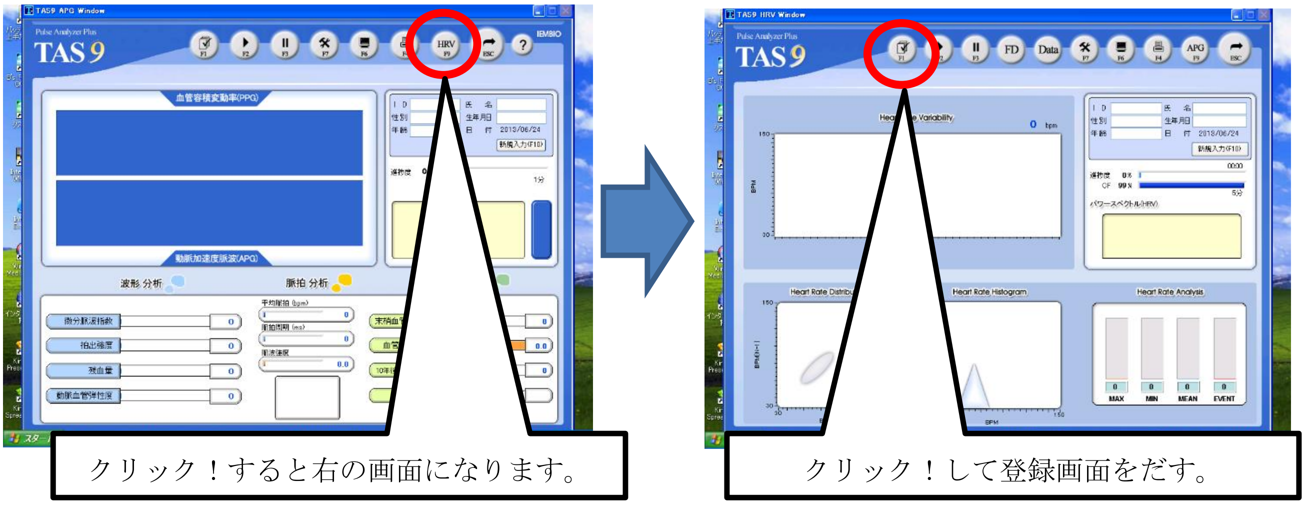

※登録画面

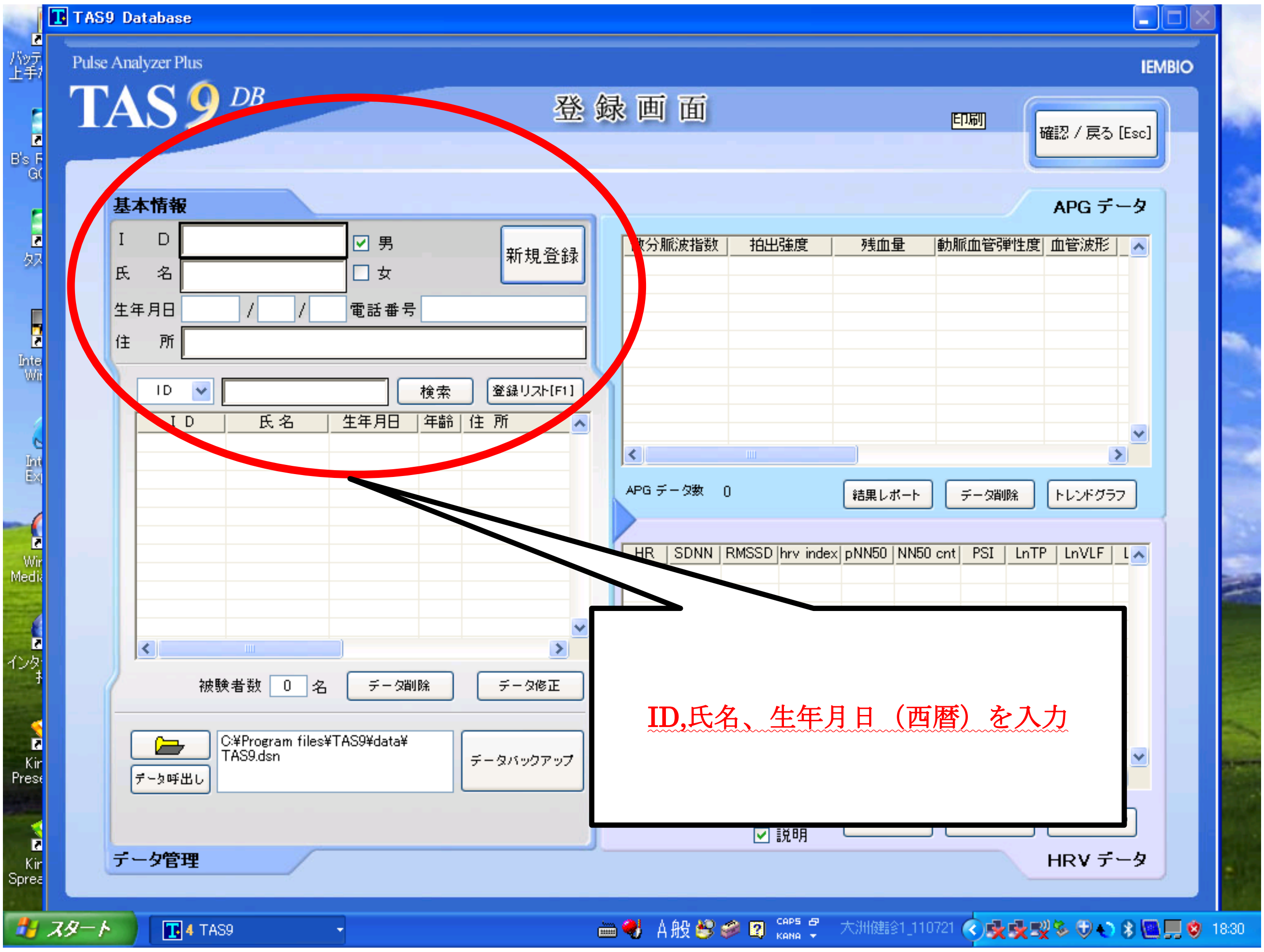

⑥登録画面で、ID（受付番号）,氏名、生年月日（西暦）を必ず入力し、「新規登録」をクリックする。登録が完了すれば、右上の「確認/戻る」をクリックする。

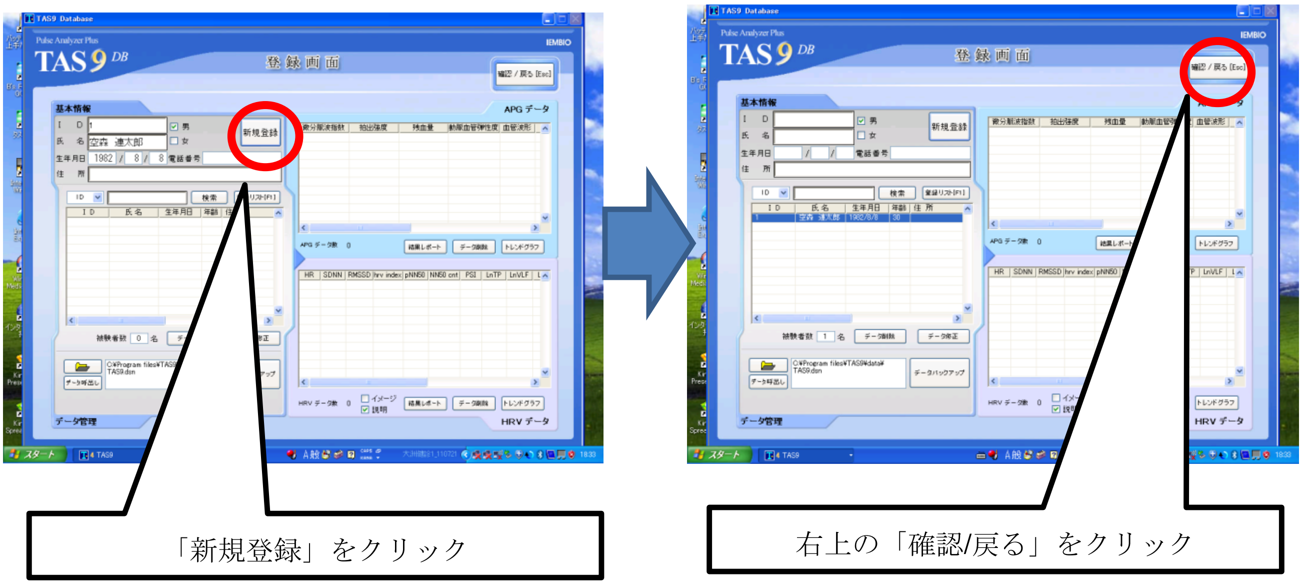

⑦被験者の示指にクリップをはめ（示指の爪甲側に赤外線センサーが来るように挟む）、座位にて安静にしてもらう。測定前に以下の注意事項を必ず伝え、「F2」をクリックして測定をスタートする。

※注意事項！！！

・「この検査の測定には５分かかります。」

・「結果を正しく出すために、測定中は安静にしていてください。体を動かしたり、しゃべったりするだけで結果が大きく変わってしまいます。」

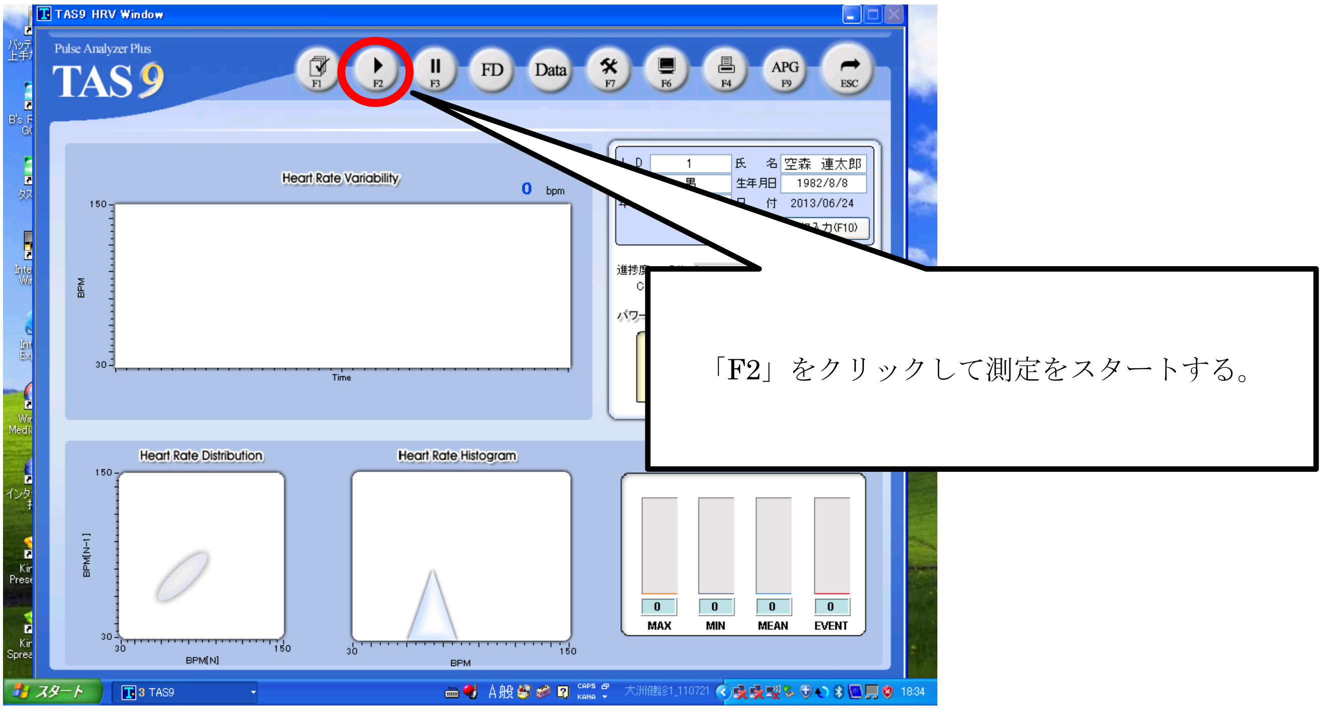

⑧測定中は安静にしてもらうように注意する。（測定中の画面）

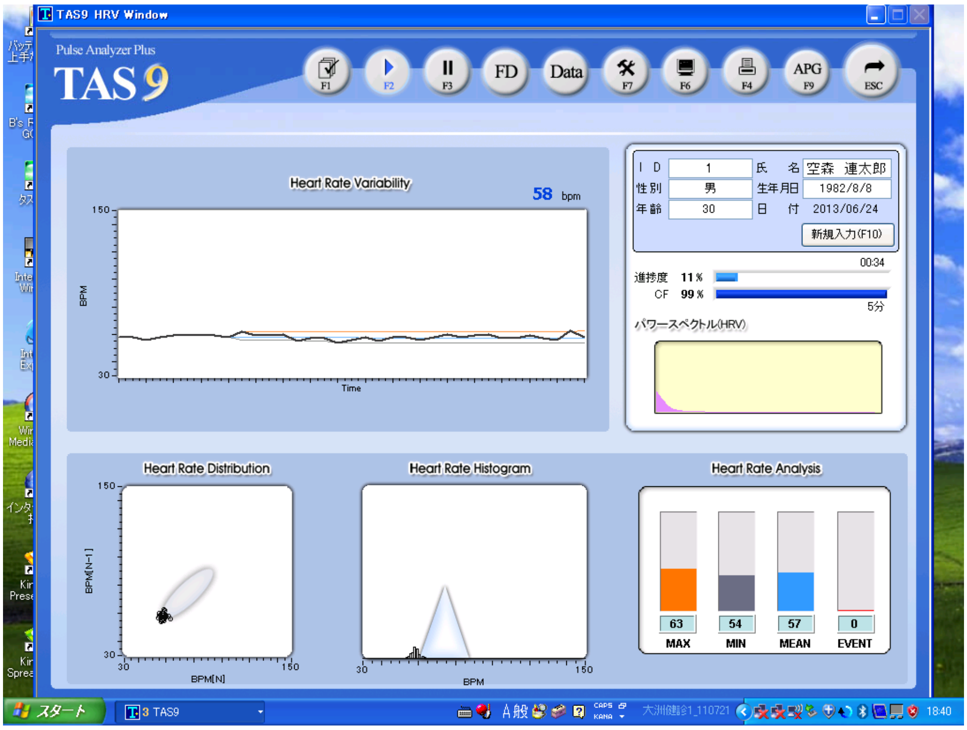

⑨測定が終了したら、「終了しました」と画面にでるので「はい」をクリックする。そして、「F6」をクリックして、結果表のプレビューを表示する。

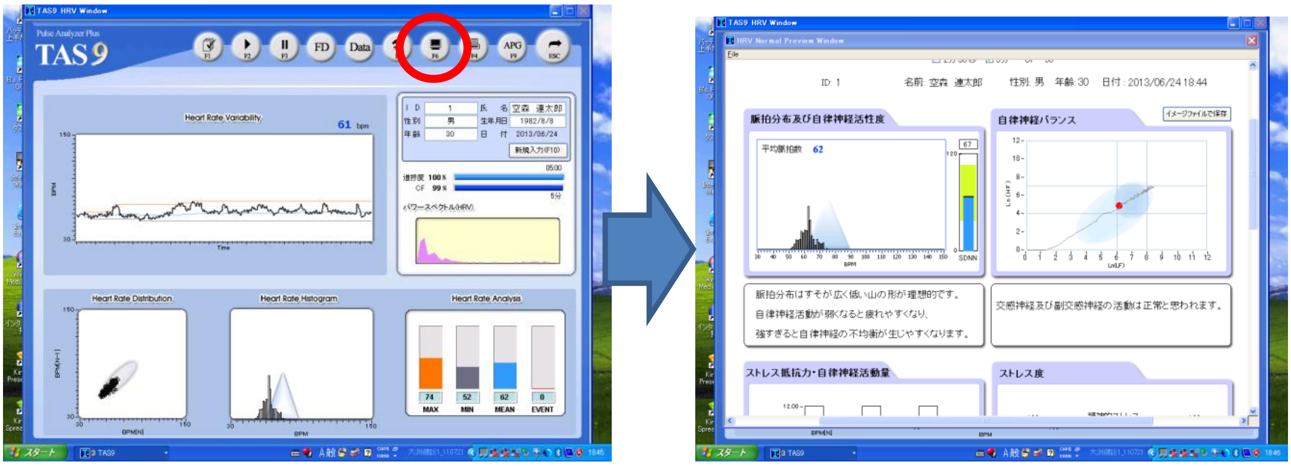

⑩プレビュー画面の左上の「File」をクリックし、「Print Window」をクリックして印刷する。印刷したものは参加希望者に渡す。TAS9 の結果用紙についてはコピー不要。PC にデータが残ります。

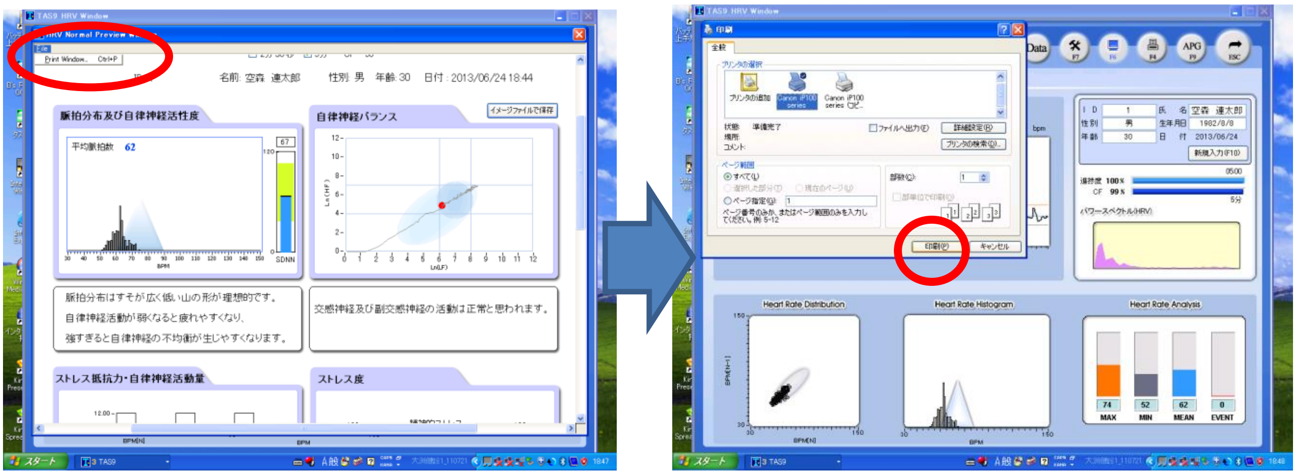

※印刷時の注意

・各 PC のプリンターの設定はさわらない。

・インクがなくなれば、別途資料「インクの換え方」を参照して換える。

⑪プレビュー画面を閉じ、「F1」をクリックして⑥～⑩を繰り返す。

データの保存について

①登録画面のデータは、基本的に「データ削除」を押さない限り、TAS９のソフトを閉じても保存されています。また、バックアップをとるとそれまでのデータが登録画面から消えてしまうので、７月 22 日の受付最終日までバックアップもとらない。受け付け順に、⑥~⑩を繰り返す。

22 日に、登録画面の「データバックアップ」をクリックし、データのバックアップをデスクトップ上に保存する。

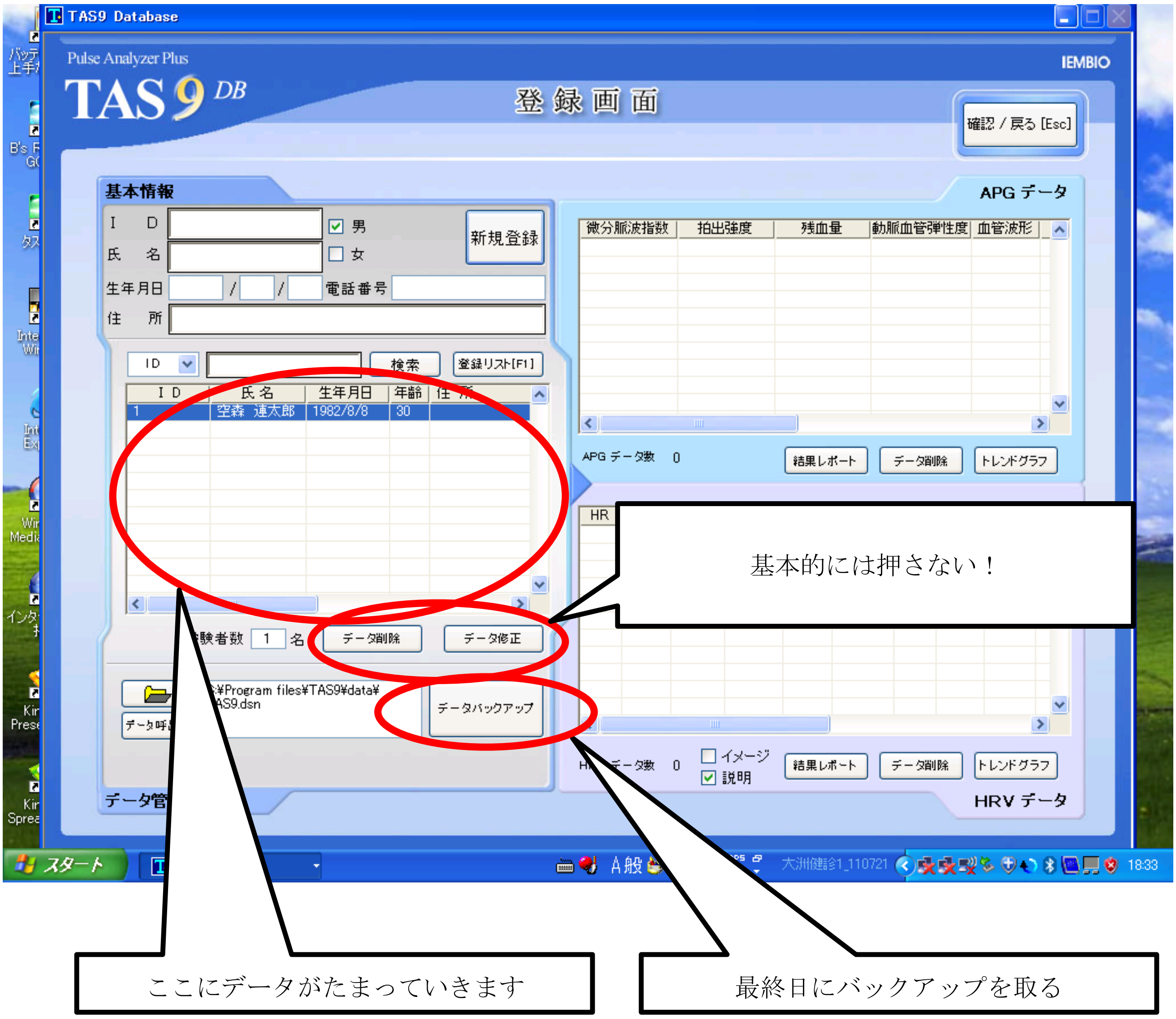

アロマセミナー実施概要

2013/07/22

【日時】8 月 1 日（木）　10：00～13：00（予定）

【場所】そらともり宴会場

【参加費】無料

【参加予定人数】60 名（予定）※本来 80 名だが申し込み状況により変更

【スタッフ】

・愛媛大学：江口依里、友岡清秀、秋元真穂、荒井夏海、伊藤亜由美、勝部璃子、本坊由華子、矢野晶子、吉原朋子（欠席：青野仁美）

・REN：後藤社長、清水マネージャー、北林先生（その他要確認）

【主な実施内容】

・研究の概要について説明

・同意書の記入

・アロマセミナー

・B コースのみ検査

・質問紙の回収（A、B コース）

【スケジュール】

	時間	内容	備考	担当
7/31	夕方？	機材搬入	TAS9、血圧計、アラ元気、良導絡をそらともりに搬入。前日までに教室から搬出。搬入は当日朝でも可。	全員
8/1	9：00	スタッフ集合	現地集合	全員
	9：30	受付開始	質問紙の回収と資料を配布。B コースには検査用紙と唾液の検査キットも渡す。<br>質問紙はすぐに記入漏れのチェック。セミナー開始まで時間があればそこで確認。特に A コースの人にはセミナー終了までに必ず確認する。	誘導×2<br>受付×2<br>質問紙×2<br>会場内誘導×2
	10：00	セミナー開始	開会のあいさつ及び研究概要の説明	江口
	10：15		同意書記入と回収	全員
	10：30		アロマセミナー	北林
	11：30	アロマセミナー終了	質問紙の不備に対応。また、A コースには 8/6 に来てもらうことを伝えて解散	
		休憩	B コースの人には紙コップを渡し、うがいをしてもらう。	
	11：40	検査	B コースのみ（血圧、TAS9、アラ元気、良導絡）	
	13：00	検査終了	終了次第、順次解散。9/3 の中間検査および介入に必ず来てもらよう伝える。	

【会場見取り図】

■宴会場まで

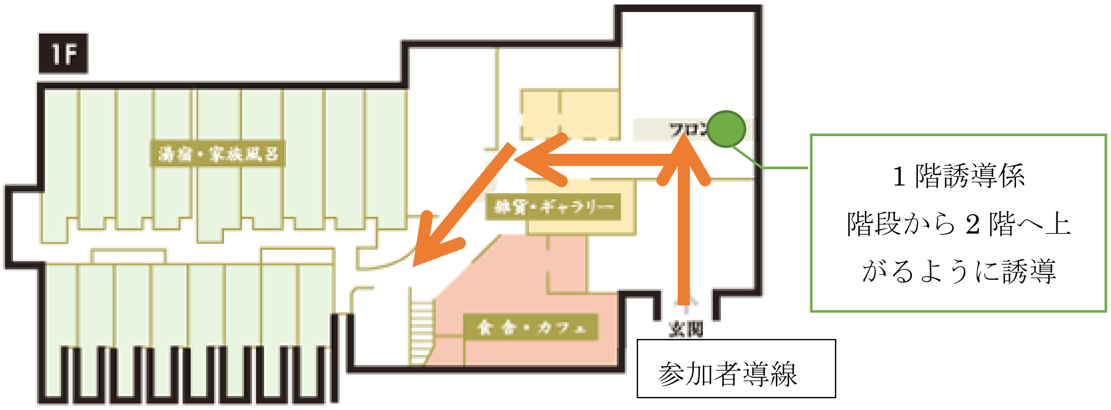

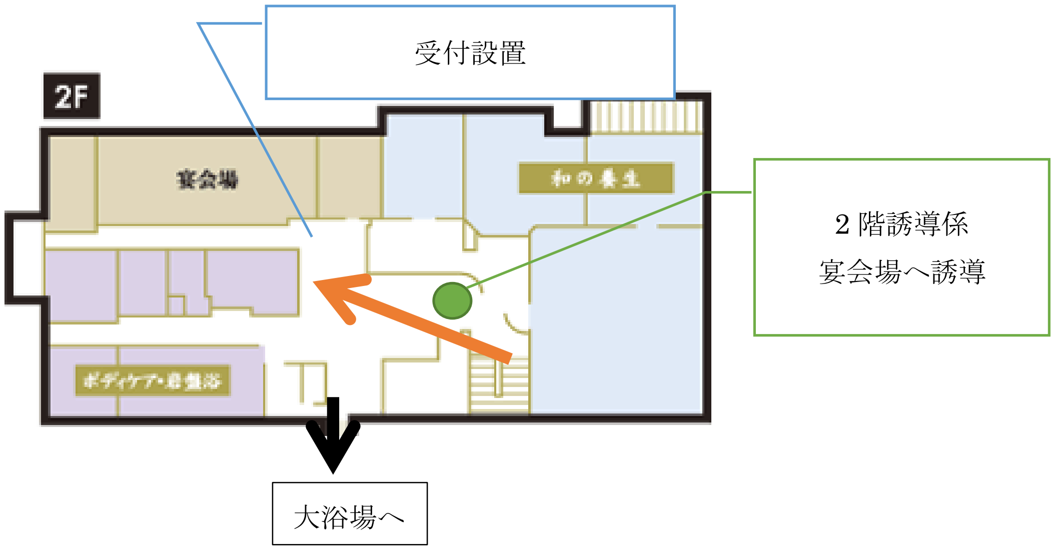

■宴会場

※人数次第だが、宴会場を全部使えるか、半分だけかは要確認。できれば全部使えて下記のように前後に講演スペースと検査スペースを分けて配置できればベスト。座席数については申し込み状況により変更。

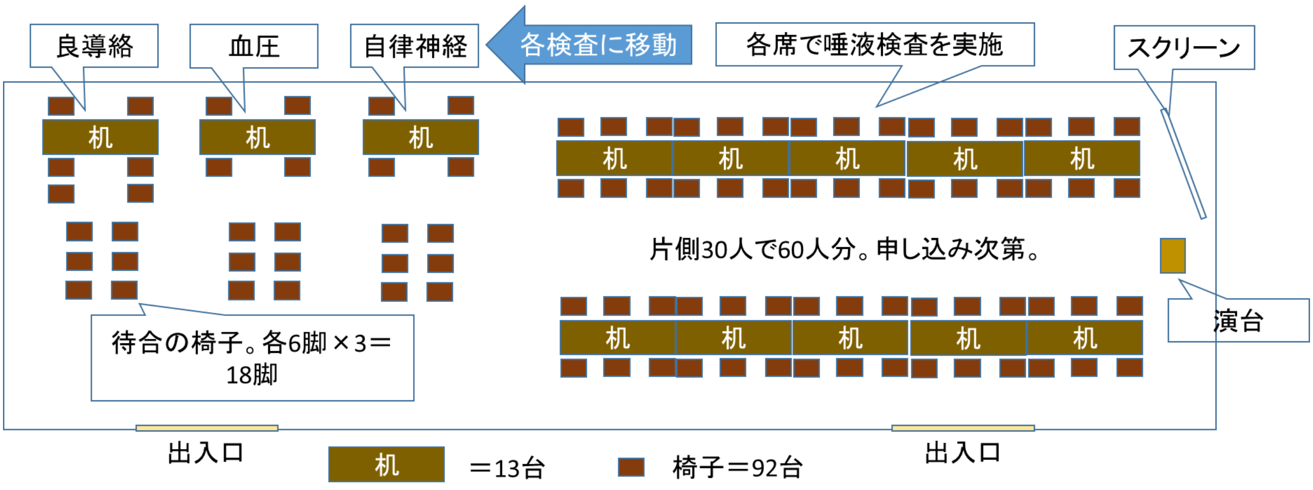

【検査の流れ】

・検査項目：自律神経機能検査、血圧測定、唾液検査、良導絡

・唾液検査を一斉に実施⇒自律神経、血圧、良導絡に均等に配置されるように誘導し、後は空いているところに行ってもらう。

【役割分標】

	担当	人数	担当
受付・誘導	会場外誘導	2	吉原、伊藤
	受付	2	勝部、荒井
	会場内誘導	2or1	矢野
	質問紙	2or3	本坊、秋元
セミナー	司会進行	1	友岡
	研究説明	1	江口
検査	誘導	1	荒井
	自律神経	1or2	勝部
	血圧	1or2	本坊
	唾液	1	吉原
	良導絡測定	2	友岡、村越
	良導絡記録	2	秋元
	確認受付	1	伊藤

【準備するもの】(※参加人数により変更有)

	用意するもの（個数※参加者60人の場合） ＊はそらともりものを使用
会場	椅子＊（89）、机＊（13）、プロジェクター＊（1）、スクリーン＊（1）、マイク＊（1）、PC（1）
受付・誘導	受け付け名簿（1）<br>誘導用の看板（3）<br>筆記用具（60本）、付箋（適宜）<br>資料（説明文書、同意書、アロマの資料（あれば））（60）
検査	検査用紙（30）<br>血圧計（3）<br>TAS９（3）※あらかじめ名簿を入力しておく<br>アラ元気（1）<br>綿棒（30）<br>精製水（1）※<br>ジップロック（30）※<br>紙コップ（30）※<br>良導絡（2）<br>延長コード（3）<br>養生テープ（1）<br>各検査の看板（3）<br>※は事前に購入の必要あり

参加者の皆様へ

『ホットスタジオにおけるアロママッサージによる心理的及び身体的影響』

1. 本研究の背景

アロマテラピーに関する身体的、心理的な効果が明らかになっています。最近では、これらのリラクセーション効果が注目され、フィットネスクラブ等の様々なプログラムの中で体験できるようになっています。しかしながら、長期的な効果等、まだまだ未解明なことがあります。

2. 本研究の目的

今回は、皆様に「そらともり REN」の人気プログラムである「身美温癒」にてホットスタジオにおけるアロママッサージを体験していただき、自律神経や血圧、ストレス度等の体や精神状態に及ぼす影響を明らかにすることを目的としています。

3. 本研究の方法

1）研究の内容

期間：2013 年 8 月 1 日（木）～9 月 29 日（日）

※8 月 1 日（木）は研究説明会およびアロマセミナーを行います。必ずご参加ください。

対象：２０歳～７０歳の男女

※対象は抽選にて決定します。ご希望に添えないことがありますことをご了承下さい。

内容：ホットスタジオにおけるアロママッサージを体験していただき、健康に関する効果を体験していただきます。

定員：約８０名

※以下の方は研究に参加することができません。

・重度高血圧（収縮期血圧 180mmHg 以上、拡張期血圧 110mmHg 以上）、その他狭心症などの循環器疾患を持つ者。

・安静時の脈拍が、110 拍/分以上、または 50 拍/分以下の者。

・重症不整脈をもつ者。

・妊娠中の者

・その他（運動中止と判断された者）

本研究に参加される方には２つのグループに分かれていただきプログラムに参加した群としない群についてプログラムの効果を比較します。グループ分けは無作為に行いますので、どちらのグループになるかは分かりません。

・A コース（40 人）：8 月に「身美温癒」 に週 3 回計 12 回参加。9 月は「身美温癒」をお休みいただきます。

・B コース（40 人）：9 月に「身美温癒」に週 3 回計 12 回参加。8 月は「身美温癒」をお休みいただきます。

2）研究の流れ
以下の流れで行われます。

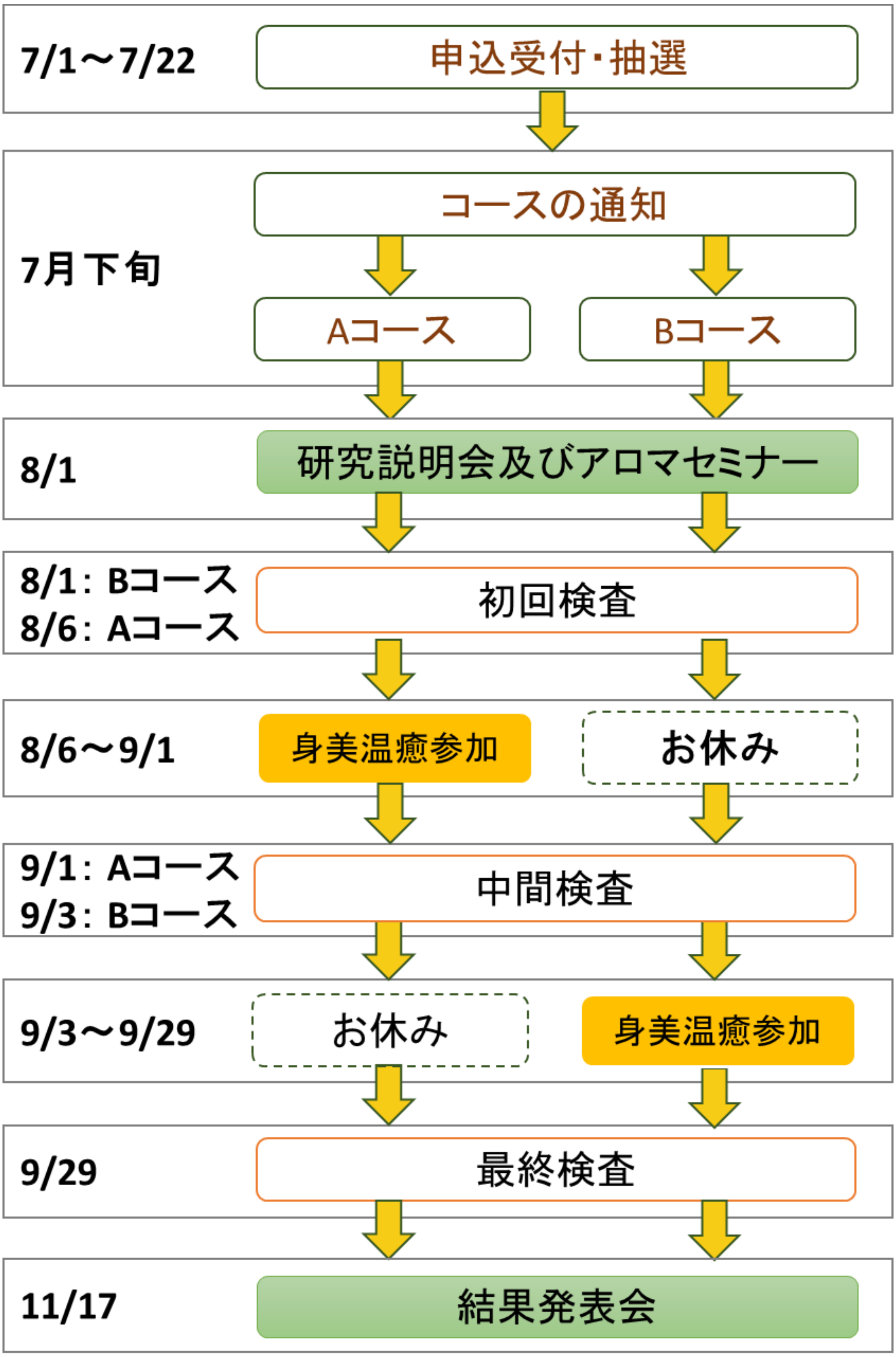

A コース

① 各種検査を実施します。
② その後、週 3 回、1 ヶ月間、計 12 回にわたり「身美温癒」のプログラムに参加します。
③ 第 1 回、第 12 回のプログラムの前後には、血圧、自律神経機能、唾液検査、東洋医学検査を実施します。
④ 12 回のプログラム終了後、各種検査を実施します。
⑤ その後 1 か月間、「身美温癒」をお休みいただき、9 月 29 日（日）に検査を受けます。

B コース

① 各種検査を実施します。
② その後 1 ヶ月間「身美温癒」のプログラムはお休みです。
③ 1 か月後、検査を実施します。
④ その後、週 3 回、1 ヶ月間、計 12 回にわたり「身美温癒」のプログラムに参加します。
⑤ 第 1 回、第 12 回のプログラムの前後には、血圧、自律神経機能、唾液検査、東洋医学検査を実施します。
⑥ 12 回のプログラム終了後、各種検査を実施します。

研究日程

A コース		B コース	
8/1（木）　研究説明会及びアロマセミナー			
初回検査	8/6（火）	初回検査	8/1(木)
第 1 回	8/6（火）	中間検査	9/3（火）
第 2 回	8/8（木）	第 1 回	9/3（火）
第 3 回	8/11（日）	第 2 回	9/5（木）
第 4 回	8/13（火）	第 3 回	9/8（日）
第 5 回	8/15（木）	第 4 回	9/10（火）
第 6 回	8/18（日）	第 5 回	9/12（木）
第 7 回	8/20（火）	第 6 回	9/15（日）
第 8 回	8/22（木）	第 7 回	9/17（火）
第 9 回	8/25（日）	第 8 回	9/19（木）
第 10 回	8/27（火）	第 9 回	9/22（日）
第 11 回	8/29（木）	第 10 回	9/24（火）
第 12 回	9/1（日）	第 11 回	9/26（木）
中間検査	9/1（日）	第 12 回	9/29（日）
最終検査	9/29（日）	最終検査	9/29（日）
11 月中旬　結果発表会			

クラス	
火曜日	17：00-17：45
木曜日	
日曜日	9：00-9：45

※本研究参加者の身美温癒のクラスは基本的には上記時間のみとのります。

＊初回検査および中間検査の日程は各コースによって異なりますのでご注意ください。
＊8 月 1 日の研究説明会およびアロマセミナーの内容については別紙をご参照ください。

3）検査の内容

本研究は以下の検査を行います

○生理指標、

①血圧測定　　　最大／最小血圧、脈拍

②自律神経機能測定

加速度脈波測定器によるHRV（Heart Rate Variability ： 心拍間変異分析）

③体成分分析

細胞内水分、細胞外水分、タンパク質、ミネラル、体脂肪量、骨格筋量、筋肉量、除脂肪量、体重、BMI、体脂肪率、ウエストヒップ比（WHR)、部位別筋肉量、部位別筋肉率、内臓脂肪レベル、腹囲、ECW/TBW(細胞外水分比)、栄養評価、身体バランス、身体強度、適正体重、体重調節量、脂肪調節量、筋肉調節量、フィットネススコア、研究項目（Obesity Degree、BCM、BMC、BMR、AC、AMC、Waist)、体成分累積結果（10回測定結果)、部位別・周波数別インピーダンス提供

④唾液検査（酸化還元確認計「アラ！元気」）

唾液を綿棒に含ませて、測定器に装着するだけで、１分以内で直ぐに測定値が表示され、その数値で、唾液が酸化又は還元されているかが確認できます。

⑤東洋医学検査（良導絡）

良導絡は東洋医学と西洋医学が融合した測定システムです。手足24か所のツボに微弱電流を流すことで、症状、元気度、ストレス状態などがわかります。

○質問紙調査

①生活習慣（運動習慣、食生活、飲酒、喫煙、睡眠）

②自覚的ストレス（質問紙）

③健康関連QOL（SF-8質問紙にて８つの下位尺度から測定）

④不安状態（STAI質問紙にて状況不安、特性不安を測定）

⑤気分や感情の状態（POMS質問紙にて測定）

4）実施場所　　　松山市南久米町「そらともりＲＥＮ」にて実施します。

4. 本研究によって予期される効果

アロマテラピーや足浴によりリラクセーション効果が期待できます。血圧値の低下、自律神経機能の向上、ストレスの低下等が期待できます。また、本研究に参加することで、さまざまな身体的・精神的な検査とその結果の通知を無料で受けることができます。

5. 本研究によって予期される副作用

本研究にて使用するアロマによるフットマッサージにて、これまでに副作用などの不利益は報告されていません。検査も侵襲性は低く、これまでに合併症などは報告されていません。

6. 本研究以外の治療法の有無及びその内容

特にありません。

7.本研究に同意しなくても治療上の不利益を受けない

本研究への参加はあなたの自由意思によるものであり、もし同意しなくてもそれによる不利益は何ら被ることはありません。

8. 本研究に同意した場合であっても，随時これを撤回することができる

この研究への参加はあなたの自由意思によるものです。この同意はいつでも撤回することができます。

9.この研究中に、あなたの健康に被害が生じた場合について

「そらともりＲＥＮ」は団体保険に加入しています。施設内において障害が発生した場合には速やかに対応し、必要な場合には保険の範囲内で治療を行います。

10.費用負担について

本研究で行う検査について、一切の費用負担は発生しません。但し、研究期間中に新規入会される方の会費については各自の負担とさせていただきます。

11.参加者の人権が厳重に保障される

・個人の結果について

個人の検査結果については、結果説明会を行い参加者の皆様に個別で通知します。

・全体の結果について

研究終了後に参加者全員を対象にした結果説明会を実施し、全体の結果をご報告します。

本研究により収集されたデータは、全て匿名化し個人を特定できない形で、解析および保存されます。学会や論文などで本研究の結果を発表する際にも、個人を特定できる形での発表は行いません。また、参加者の皆様が本研究の結果を知りたい場合、他の参加者の個人情報や本研究の独創性に支障がない範囲内で、方法や結果についてお伝えします。

12.本研究について質問あるいは疑問がある時は，担当者に尋ねることができること

本研究について、あなたの健康上、質問などがある時には、担当者に尋ねることができます。また、本研究により、あなたの健康上、必要かつ新たな情報が得られた場合は速やかにご連絡をいたします。

13.研究実施講座名および責任医師名、連絡先など

愛媛大学大学院医学系研究科公衆衛生・健康医学　教授　谷川武　担当　江口・友岡

〒791-0295 愛媛県東温市志津川 / TEL 089-960-5283 / FAX 089-960-5284

※夜間連絡先

そらともり REN

〒790-0924 愛媛県松山市南久米町 243 番地 1/TEL 089-976-3086/FAX 089-976-3085

同　意　書

愛 媛 大 学 医 学 部 附 属 病 院 長　殿
愛媛大学大学院医学系研究科公衆衛生・健康医学　教授殿

私は、担当者から、「足浴と温熱環境におけるアロママッサージによるリラクセーション効果に関する心理的及び肉体的影響についての比較研究」の目的と方法、予期される効果及び副作用、他の治療方法の有無及び内容などについて文書により十分説明を受け理解しました。

今回の研究に参加するのは自由意志に基づくものであり、いつでもこれを撤回し、研究の中止を申し出ることができること、同意しないあるいは撤回した場合でも不利益を受けないこと、人権が厳重に保障されることを理解しましたので、本研究に参加することに同意します。

【ご本人】
同意年月日　平成　　年　　月　　日　署　名＿＿＿＿＿＿＿＿＿＿

生年月日　明治・大正・昭和　　年　　月　　日

【代諾者】
同意年月日　平成　　年　　月　　日　署　名＿＿＿＿＿＿＿＿＿＿

ご本人との関係＿＿＿＿＿＿＿＿＿＿

【立会人】

立会年月日　平成　　年　　月　　日　署　名＿＿＿＿＿＿＿＿＿＿

【説明者（主担当者・分担者）】
説明日　平成　　年　　月　　日　＿＿＿＿＿＿科（部）　署名＿＿＿＿＿＿＿＿

※補足的な説明を行った場合は下記に記入
【補足説明者】
説明日　平成　　年　　月　　日　＿＿＿＿＿＿科（部）　署名＿＿＿＿＿＿＿＿

同　意　撤　回　書

愛 媛 大 学 医 学 部 附 属 病 院 長　殿

愛媛大学大学院医学系研究科公衆衛生・健康医学教授殿

　私は、「足浴と温熱環境におけるアロママッサージによるリラクセーション効果に関する心理的及び肉体的影響についての比較研究」について説明を受け、平成 24 年 8 月 9 日に本研究に参加することに同意しましたが、これを撤回します。

【ご本人】

平成　　　年　　　月　　　日　署　名＿＿＿＿＿＿＿＿＿＿＿＿

生年月日　明治・大正・昭和　　　　年　　　月　　　日

【代諾者】

平成　　　年　　　月　　　日　署　名＿＿＿＿＿＿＿＿＿＿＿＿

ご本人との関係＿＿＿＿＿＿＿＿

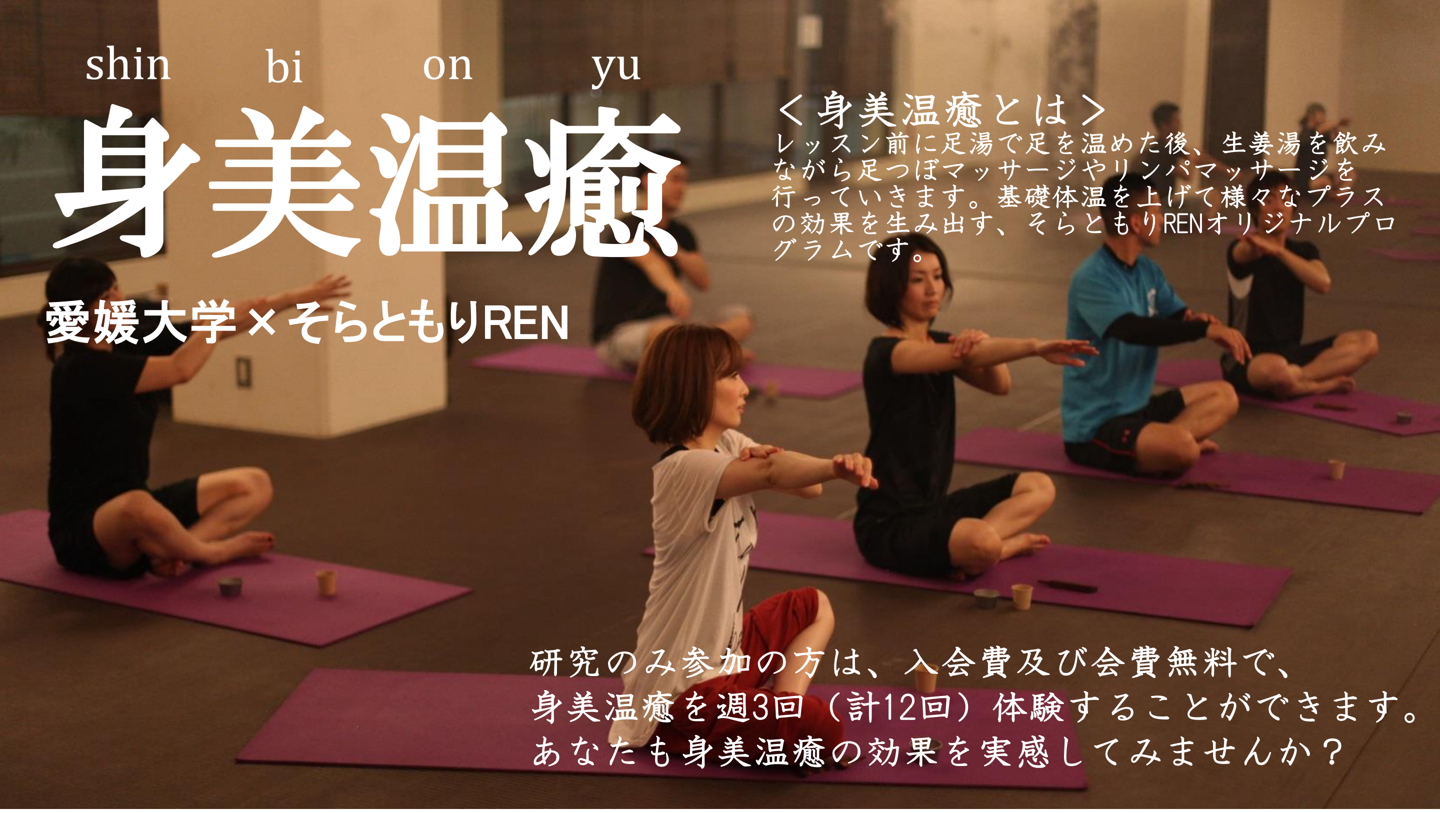

shin　bi　on　yu

身美温癒

愛媛大学×そらともりREN

＜身美温癒とは＞
レッスン前に足湯で足を温めた後、生姜湯を飲みながら足つぼマッサージやリンパマッサージを行っていきます。基礎体温を上げて様々なプラスの効果を生み出す、そらともりRENオリジナルプログラムです。

研究のみ参加の方は、入会費及び会費無料で、
身美温癒を週3回（計12回）体験することができます。
あなたも身美温癒の効果を実感してみませんか？

研究参加者募集中

身美温癒の医学的効果に関する研究を今年も継続して行うことになりました。今年は特に、自律神経機能の改善に注目し、アロマオイルも特別に調合したものを使用します。アロマセラピーやマッサージは自律神経機能の改善に有効といわれており、身美温癒もその効果が大きく期待できます。
皆様の奮ってのご参加をお待ちしております。

自律神経機能検査、唾液によるストレス検査、
東洋医学的検査など様々な検査を受けることができ、
自分の健康状態を詳しく知ることができます。

＜検査内容＞
自律神経機能検査、血圧、良導絡、唾液検査、Inbody(体成分分析)、
生活習慣・睡眠・主観的健康観やストレスに関する質問項目
※申し込み受付時に自律神経機能、血圧測定、Inbodyを無料で実施いたします。

■申し込み期間
2013年7月1日（月）〜7月22日（月）

■対象　20歳〜70歳の男女
※重度高血圧,重度不整脈,妊娠中の方,その他重度の疾患のある方は参加できません。
※会員･非会員ともに参加できます。
※申し込み多数の場合、対象は抽選にて決定します。参加ご希望に添えないことがありますことをご了承ください。

■参加費　無料
※研究のみ参加の方は、入会費及び会費はかかりません。
※研究に参加される方で新規入会する場合、入会金が免除になります。（11月30日迄）

■定員 80名

■参加条件
8月または9月の1か月間、週3回、下記の時間帯に身美温癒を必ず受けることができるもの。

火曜日	17:00〜17:45
木曜日	17:00〜17:45
日曜日	9:00〜9:45

■主催
愛媛大学大学院医学系研究科公衆衛生
そらともりREN

■申し込み方法
そらともりRENにて受付中
※受付時に自律神経機能及び血圧測定を無料で実施いたします。

参加希望、その他ご質問などお気軽にお問い合わせください。

REN Sora to Mori
営業時間　6:00〜24:00

そらともりREN
愛媛県松山市 南久米町243番地1
TEL. 089-976-3086

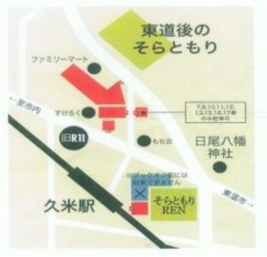

コースについて

参加者の皆さまには、A,Bの二つのコースに分かれていただきます。AコースとBコースの振り分けはこちらで行います。（コースのご希望には添えませんのでご了承ください。）

8/1～9/29 の2か月間研究を実施します。

<u>○Aコース</u>
・8/6～9/1に「身美温癒」に週3回計12回参加
・9/2～9/29は「身美温癒」をお休み
・8/6に初回検査、9/1に中間検査、9/29に最終検査を実施

<u>○Bコース</u>
・9/3～9/29に「身美温癒」に週3回計12回参加
・8/6～9/1は「身美温癒」をお休み
・8/1に初回検査、9/3に中間検査、9/29に最終検査を実施

<u>※両コースとも8/1のアロマセミナーにはご出席ください。</u>

※「身美温癒」以外のプログラムの利用を希望する方は、ご入会下さい。（ただし、お休み期間中の「身美温癒」のご利用はご遠慮ください）

コースの流れ

時期	Aコース	Bコース
7/1～7/22	申込受付・抽選	
7月下旬	コースの通知	
	Aコース	Bコース
8/1	研究説明会及びアロマセミナー	
8/1：Bコース<br>8/6：Aコース	初回検査	
8/6～9/1	身美温癒参加	お休み
9/1：Aコース<br>9/3：Bコース	中間検査	
9/3～9/29	お休み	身美温癒参加
9/29	最終検査	
11月中旬	結果発表会	

スケジュール

Aコース		Bコース	
アロマセミナー　8/1（木）			
初回検査	8/6（火）	初回検査	8/1（木）
第1回	8/6（火）	中間検査	9/3（火）
第2回	8/8（木）	第1回	9/3（火）
第3回	8/11（日）	第2回	9/5（木）
第4回	8/13（火）	第3回	9/8（日）
第5回	8/15（木）	第4回	9/10（火）
第6回	8/18（日）	第5回	9/12（木）
第7回	8/20（火）	第6回	9/15（日）
第8回	8/22（木）	第7回	9/17（火）
第9回	8/25（日）	第8回	9/19（木）
第10回	8/27（火）	第9回	9/22（日）
第11回	8/29（木）	第10回	9/24（火）
第12回	9/1（日）	第11回	9/26（木）
中間検査	9/1（日）	第12回	9/29（日）
最終検査	9/29（日）	最終検査	9/29（日）
11月中旬　結果発表会			

クラス	
火曜日	17：00-17：45
木曜日	17：00-17：45
日曜日	9：00-9：45

※できるだけ全日程にご参加ください。
※赤字は検査日になります。必ずご参加ください。

注意事項

■各コースで初回、中間の検査日が異なります。
■検査日は集合時間が異なります。詳細は追ってご連絡いたします。
■レッスン開始20分前までに受け付けをお済ませください。
■タオル、ペットボトルは貸し出します。
■着替えの貸し出しは行いません。Tシャツ、短パン、下着などはご持参ください。
■研究のみご参加の方（非会員）の入浴施設の利用はご遠慮ください。
